# Targeted co-expression networks for the study of traits

**DOI:** 10.1038/s41598-024-67329-7

**Published:** 2024-07-19

**Authors:** A. Gómez-Pascual, G. Rocamora-Pérez, L. Ibanez, J. A. Botía

**Affiliations:** 1 Communications Engineering and Information Department, University of Murcia, 30100 Murcia, Spain; 2 Department of Genetics and Genomic Medicine Research and Teaching, UCL GOS Institute of Child Health, London, WC1N 1EH UK; 3 Department of Psychiatry, Washington University School of Medicine, Saint Louis, MO 63110 USA; 4 Department of Neurology, Washington University School of Medicine, Saint Louis, MO 63110 USA

**Keywords:** Co-expression, Genes, LASSO, Trait, WGCNA, Computational biology and bioinformatics, Computational models, Gene regulatory networks, Alzheimer's disease

## Abstract

Weighted Gene Co-expression Network Analysis (WGCNA) is a widely used approach for the generation of gene co-expression networks. However, networks generated with this tool usually create large modules with a large set of functional annotations hard to decipher. We have developed TGCN, a new method to create Targeted Gene Co-expression Networks. This method identifies the transcripts that best predict the trait of interest based on gene expression using a refinement of the LASSO regression. Then, it builds the co-expression modules around those transcripts. Algorithm properties were characterized using the expression of 13 brain regions from the Genotype-Tissue Expression project. When comparing our method with WGCNA, TGCN networks lead to more precise modules that have more specific and yet rich biological meaning. Then, we illustrate its applicability by creating an *APP*-TGCN on The Religious Orders Study and Memory and Aging Project dataset, aiming to identify the molecular pathways specifically associated with *APP* role in Alzheimer’s disease. Main biological findings were further validated in two independent cohorts. In conclusion, we provide a new framework that serves to create targeted networks that are smaller, biologically relevant and useful in high throughput hypothesis driven research. The TGCN R package is available on Github: https://github.com/aliciagp/TGCN.

## Introduction

Gene co-expression networks (GCNs) provide a framework to organize, integrate and analyze large-scale transcriptomic data sets^1^. To create gene co-expression networks, different methods have been proposed. One of those approaches for GCN construction is based on Bayesian theory^2^. Due to their probabilistic nature, these networks can identify relevant indirect interactions between genes. Still probabilistic but slightly different, we find partial-correlation-based methods such as SPACE (Sparse PArtial Correlation Estimation)^3^. These are based on undirected probabilistic graphical models that describe the conditional independence relationship among nodes (genes) under the assumption of a multivariate Gaussian distribution of the data. An alternative to probabilistic approaches are information-theory-based methods such as ARACNE (Algorithm for the Reconstruction of Accurate Cellular Networks)^4^, CLR (context likelihood of relatedness)^5^, MRNET (maximum relevance/minimum redundancy)^6^ and RELNET (relevance networks)^7^. They use the mutual information notion^8^ to identify high confidence transcriptional interactions. Some other approaches focus on the group and subgroup structure underlying the transcript expression and large networks are broken down into smaller (sub) networks (also called modules or communities), usually more amenable to biological interpretation. These community detection methods include the Combo^9^, Conclude^10^, Fast Greedy^11^, Leading Eigen^12^, Louvain^13^ and Spinglass^14^ tools. An alternative approach focuses on different information modalities, by combining individual functional association networks and composing a functional association network that summarizes all available evidence of co-functionality. An example is GeneMANIA (Multiple Association Network Integration Algorithm)^15^, which integrates multiple functional association networks used to predict gene function. Currently, the most widely used gene network is the co-expression network. Within those types of networks, WGCNA^16^ is the preferred method to create the networks using unsupervised clustering, without the use of a priori defined gene sets^17–20^. WGCNA’s clustering distance is based on the notion of adjacency, which is a correlation transformed to achieve scale-free topology networks. Moreover, the co-expression modules detected by WGCNA can be improved by an additional refinement step based on applying the k-means algorithm on those modules, and available in the CoExpNets R package^21^ we proposed. The co-expression modules that emerge from this approach are subsequently annotated through (1) their association with traits of interest, (2) their enrichment for specific cell type markers and (3) the characterization of the pathways involved in each of the modules. By using the GBA (Guilty By Association) assumption^22^, all genes in a module are said to receive the same annotation as the module as a whole. It usually implies that the identified gene modules are too large in size, due to its unsupervised approach. Therefore, downstream annotations emerging from those modules become burdensome, eg. association with several unrelated traits, enrichment for markers of diverse cell types, and a large set of functional annotations from diverse ontologies. We particularly use Gene Ontology (GO)^23^, Reactome Pathway Database (REAC)^24^, Kyoto Encyclopedia of Genes and Genomes (KEGG)^25^ and Human Phenotype Ontology (HP)^26^ as bare minimum. Taken together, it is difficult to disentangle the biological annotations that correspond to a unique trait within a single module.

To facilitate the study of single traits on high throughput transcriptomic studies, we propose to use targeted co-expression networks (TGCNs). Given a transcriptomic dataset and a trait of interest (e.g. age, disease status, even a specific gene could be such a feature), the proposed algorithm starts with selecting the most relevant transcripts to predict that trait with LASSO regression^27^ (Least Absolute Shrinkage and Selection Operator). The detected transcripts are then used as seeds of the co-expression modules in the sense that we use them to recreate the rest of the module. Those seeds are seen as single representatives of the different biological pathways related to the trait as mediated by gene expression. We then recreate those pathways by selecting, for each seed, those transcripts that are co-expressed (i.e., highly and reliably correlated) and identify the pathways and additional function with thorough annotation. TGCNs deliver a manageable number of small modules and, overall, at least one order of magnitude smaller GCN in gene size (see results) in comparison to WGCNA. Therefore, targeted GCNs are more suited for hypothesis driven research of gene expression-based studies.

This study is divided into two main sections, (i) characterization of the algorithm properties, and (ii) application to real world transcriptomic data to illustrate its utility. For the first part, we used for experiments the expression data of 13 different brain regions from the Genotype-Tissue Expression study (GTEx). We begin by testing the robustness of the transcript seed selection process and the stability of this selection. We then continue characterizing the general properties of the targeted GCNs and finish comparing them with WGCNA ones in the same sample sets from GTEx. In the second part of the paper, we illustrate the use of the proposed TGCN software with expression profiles of Alzheimer’s disease (AD) brains. Pathological hallmarks of AD include presence of intracellular hyperphosphorylated forms of the microtubule-associated protein tau (neurofibrillary tangles) and extracellular amyloid beta (Aβ) plaques^28^. The Aβ peptide results from the sequential cleavage of Amyloid Precursor Protein (the *APP* gene) by β- and γ-secretases. We hypothesize that an *APP*-targeted GCN (*APP*-TGCN) on AD brain transcriptomics can shed light on the functional characterization of *APP* in AD pathology. We used the ROSMAP brain transcriptome dataset to create an *APP*-TGCN. Our findings were further replicated in the Mayo Clinic and MSBB transcriptomic datasets.

## Results

### Bootstrapped LASSO decreases algorithm’s instability when identifying seed transcripts

**Figure 1:**
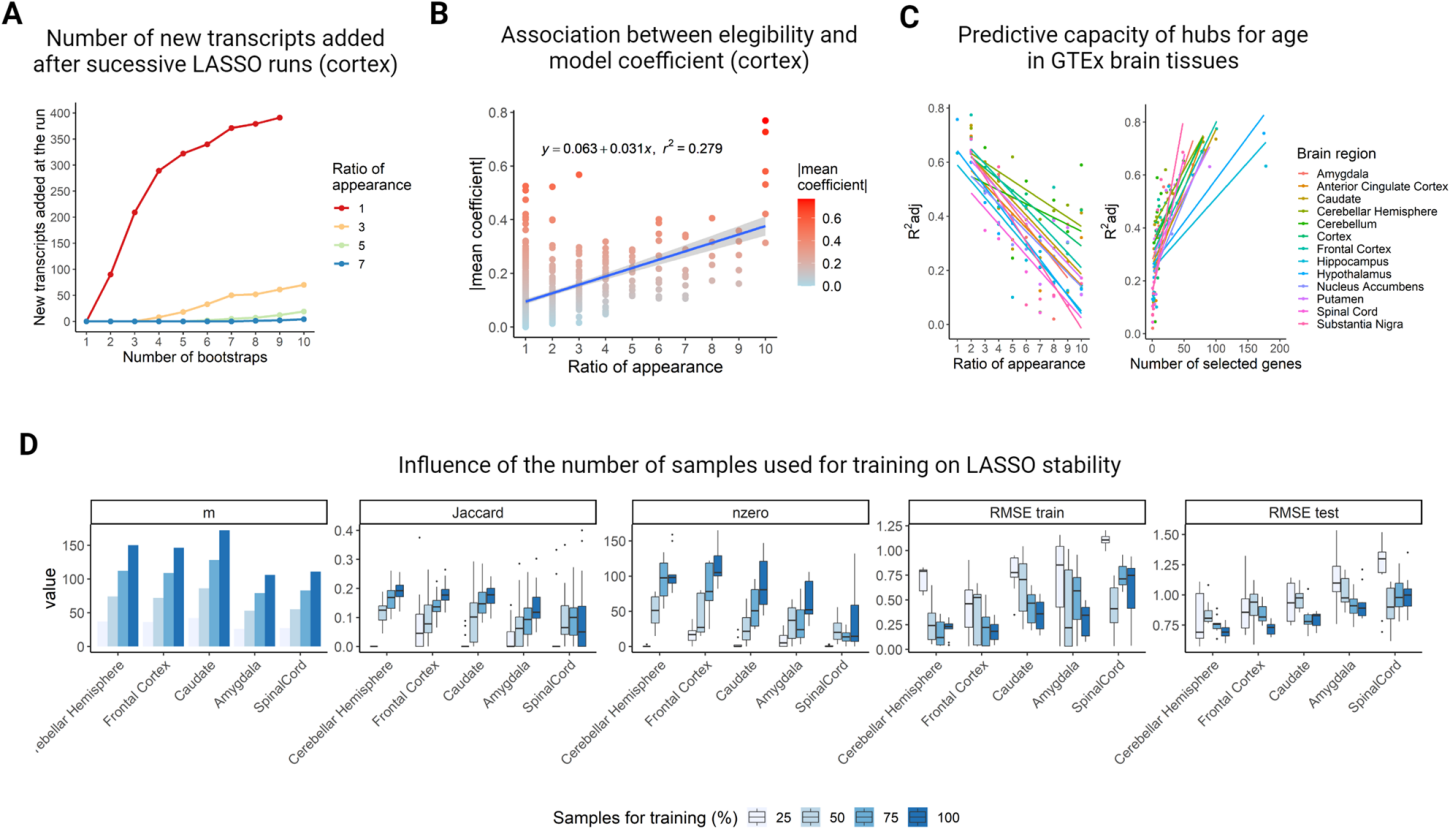
Study of lasso stability in high-dimensionality problems. (**A**) The number of new transcripts selected by a LASSO run when increasing the number of runs in comparison to only one iteration for GTEx cortex tissue with donor´s age as the predicted variable. Increasing the number of runs shows a strong increase in the number of transcripts selected at least once (red curve). We are interested in the transcripts selected by LASSO in a reasonable number of iterations, e.g. 6, 7 or 8 in the graph. (**B**) The plot shows a positive association, R^2^ 0.279 (*P* < 8.66 × 10^–38^) between the eligibility of a transcript (i.e., number of times it is selected across the *b* LASSO bootstraps) and the average size of the coefficients for that transcript within the linear models that predict the trait. This plot corresponds to the age-TGCN for GTEx cortex. Each point is a transcript, the x axis represents eligibility and the y-axis represents the mean coefficient across linear models predicting age (see supplementary table S3). (**C**) Both plots show the relation between the number of transcripts used to predict age and the adjusted R^2^ for all age-TGCNs for GTEx brain tissues. We see a clear negative relation between adjusted R^2^ at predicting age as the ratio of appearance increases (left), that is, because the number of transcripts decreases within the model (right). Plots in B and C illustrate a trade-off in using TGCNs. The larger the seed size, the better performer is the final linear model to predict the trait, but the TGCN gets also larger and difficult to interpret. (**D**) The influence of the number of samples used to train the model, *m*, is represented in terms of LASSO stability. For five different brain tissues of the GTEx dataset, 10 LASSO runs were applied using 25, 50, 75 and 100% of the samples for the training. We observe that, the higher the *m*, the higher the LASSO stability. Jaccard represents the overlap of relevant transcripts between pairs of iterations, nzero represents the number of transcripts selected through the 10 iterations and RMSE train and test represent the train and test error of the LASSO models.

The first step to generate a TGCN on a transcript expression profile is to select the transcripts that contribute to predict the trait of interest *T* with LASSO regression*.* We are concerned with the stability of LASSO when selecting the seeds. Our algorithm would be stable if a slight variation in the sample set does not significantly vary the seed transcripts set composition. To empirically assess stability, we used the gene expression profiles of 13 different brain regions from GTEx (see supplementary Table S1) to predict the age of the donors at the time of death and created 13 age-TGCNs. The empirical instability of LASSO is illustrated in Fig. 1A. Each curve corresponds to a different value of the number of times the transcripts are selected or ratio of appearance, *r*. We observe that, when the number of LASSO runs (x axis) increases, the number of seeds keeps increasing at high pace, preventing the seed geneset from becoming stable (see curve for r value of 1 in Fig. 1A). However, when we bootstrap the LASSO, then the curves stop growing early on (see curves for *r* values 6, 7 and 8 in Fig. 1A). We experimented with LASSO runs values up to 50 in all 13 brain regions and observed that, amongst all seeds detected with 10 LASSO runs, the ones that were selected in at least 75% of the models remain selected at the same rate after running 50 LASSO runs. This suggests no more than 10 bootstraps are needed. This strategy allowed us to detect the more confident and universal transcripts to predict the age of the donors in each brain region of the GTEx dataset. Additionally, we observed that, for most of the brain regions, the age-predictive models created with only the repeatedly selected transcripts as predictors (seed transcripts models) reported a lower RMSE on test compared with the LASSO models created for the same brain region (7.42 and 8.16 RMSE on test on average, respectively) (see supplementary table S2). Among the different brain regions, the highest R^2^ was observed for the cerebellum region, with a mean R^2^ of 0.732 ± 0.089, where the standard deviation represents the error of the cross validation. On the opposite side, the substantia nigra brain region reported the worst performance to predict the age of the donors, reporting a mean R^2^ of 0.193 ± 0.155. On average, the seed transcripts models reported an average R^2^ of 0.435 across the different brain regions.

Interestingly, we observe that the number of times that a transcript *t* was selected in the bootstrap is positively correlated with the magnitude for the coefficient in the LASSO models for the corresponding transcript *t* (see Fig. 1B and supplementary table S3). This suggests that bootstrapped LASSO tends to select the most relevant transcripts in the prediction of the trait. On the other hand, we empirically observe the trade-off between increasing values of $t(r)$ and lower R^2^ (Fig. 1C, left panel). Alternatively, large seed set sizes naturally increase R^2^ (Fig. 1C, right panel).

Finally, we also investigated how sample size influences stability. We use the Jaccard coefficient across runs as an estimate. Figure 1D includes experiments for brain regions that showed the highest stability (cortex), the lowest stability (spinal cord) and middle stability (caudate, hippocampus and amygdala in this order). We observe that, by increasing the training sample size, we get an increase of stability (cor = 0.535, *P* < 5.78 × 10^–62^). This trend was seen for all the explored brain regions except for the spinal cord. This tissue showed the lowest performance when predicting the age of death.

### Seed transcripts are representatives of independent pathways

**Figure 2:**
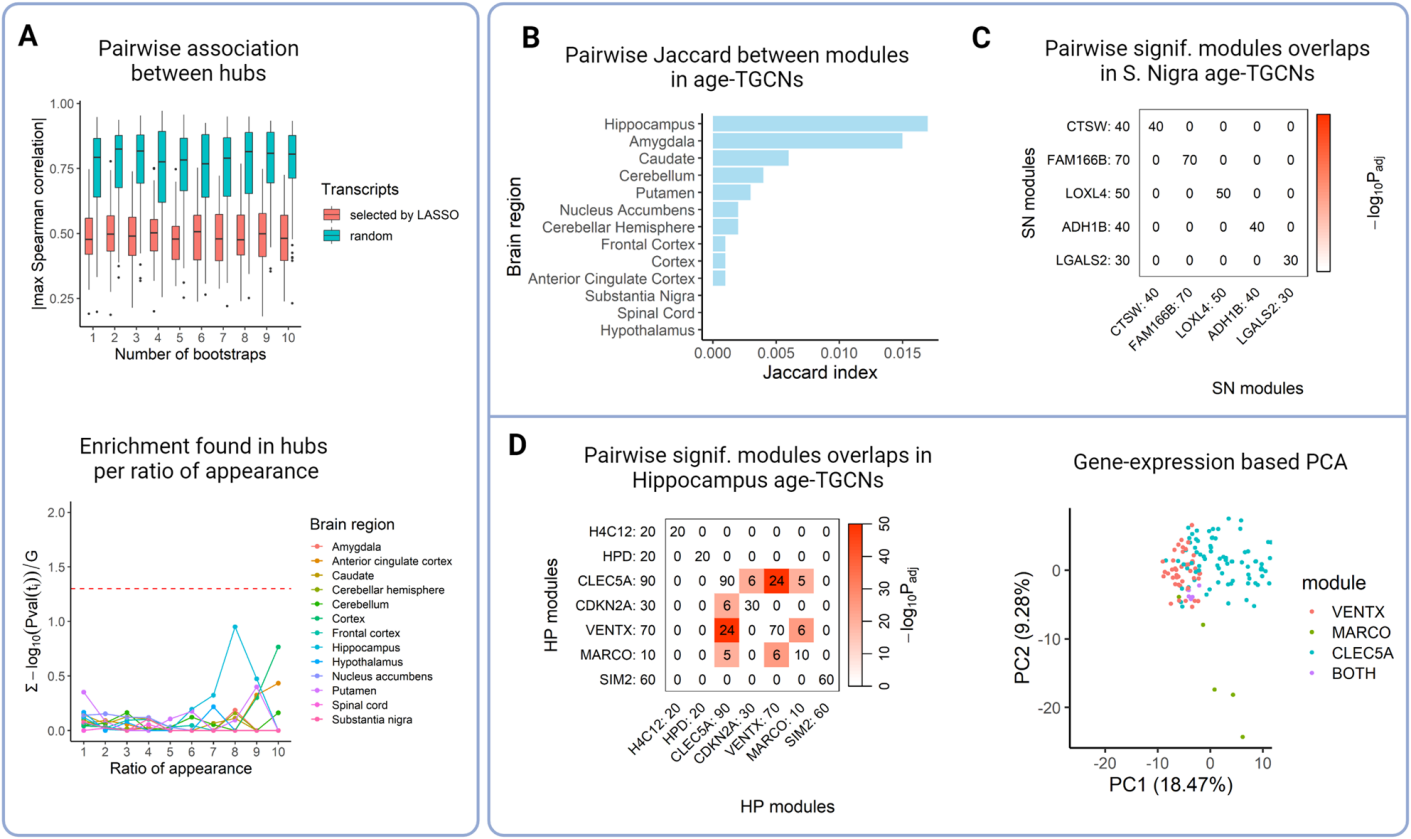
Independence of seeds and overlap across modules. (**A**) For all 13 brain tissues, we obtained the pairwise correlation between transcripts selected by LASSO (magenta) and compared them with the same number of pairs of randomly chosen transcripts (turquoise) across runs. We consistently see across runs that LASSO gets transcripts with less association (absolute value of maximum Spearman correlation between pairs) than we would expect by random chance (see supplementary table S4). This illustrates that seeds show no association and it is maintained across bootstraps. (**B**) For each brain region of GTEx, enrichment (y axis) is shown for the age-specific seed transcripts grouped per ratio of appearance (x axis). Enrichment is measured as the sum of the − log_10_P of all the annotations normalized by group size in transcripts. No geneset enrichment reaches the required cut-off for significance, − log_10_(0.05). (**C**) Average pairwise overlap between modules in all age-TGCNs from GTEx brain tissues. For all tissues, we observe an overlap, as estimated by Jaccard, close to zero. This suggests each module represents different gene functions and pathways. (**D**) Overlap between modules within the substantia nigra age-TGCN. Each row and column refer to a module, led by the corresponding seed, at the row and column label. Each cell shows the number of common transcripts. Color scale represents the adjusted -log_10_P of the overlap based on a Fisher Exact test. (**E**) Same plot as D but for the hippocampus age-TGCN. We observed that *CLEC5A* module showed a significant overlap with *CDKN2A* (*P* < 1.58 × 10^–21^), *VENTX* (*P* < 3.65 × 10^–91^) and *MARCO* (*P* < 2.01 × 10^–20^) modules with a Fisher exact test after p-value adjustment. In addition, we also observe a significant overlap between *VENTX* and *MARCO* modules (*P* < 1.40 × 10^–25^). (F) PCA projection at transcript level with the union of the transcript of modules *VENTX*, *MARCO* and *CLEC5A* from the age-targeted network of the hippocampus brain tissue. We observe that the transcripts of module *CLEC5A* go in one direction, those of *MARCO* in another, those of *VENTX* in a different direction while the transcripts common to these three modules appear at the intersection of the point clouds.

Seed transcripts are expected to be important transcripts of each pathway involved in the trait or phenotype of interest. When creating TGCNs, we implicitly assume that biological pathways influence the phenotype of interest somewhat independently and additively. Accordingly, their respective representative transcripts, the seeds, should be independent of each other. In terms of how correlated seeds are pairwise, we observe a significant difference (T-test *P* < 0.05) in the mean of the maximum pairwise correlation of LASSO selected transcripts across runs (0.489) and that observed within randomly chosen genesets paired in size (0.756) for cortex tissue from GTEx dataset (Fig. 2A, supplementary table S4, see “Methods”). Therefore, the bootstrapped LASSO identifies unassociated transcripts.

If seed transcripts represent independent pathways and we try to annotate them functionally as a geneset, we should not observe any significant enrichment. Figure 2A shows enrichment of GO, KEGG, REAC and HP terms found in seed transcripts grouped by ratio of appearance from 1 to 10. None of the seed transcript sets showed relevant functional abundance (see also methods). This shows that these transcripts are not involved in the same pathways. Therefore, they might be involved in different non overlapping pathways.

### Modules within a targeted GCN show low or null overlap

For each brain region of the GTEx dataset, the transcripts selected by LASSO in at least 8/10 iterations were kept as seed transcripts since we considered these are reliable and we also obtained a good balance between the number of modules created and the RMSE of the model (see supplementary table S2). Interestingly, we observed that the seed transcripts detected per brain region replicate, on average, in seven of the 12 different brain regions of the study (for more information, see supplementary Fig. S1).

During the process of module creation, potential module member transcripts may show high correlation with more than one seed. TGCN allows transcripts to belong to more than one module, although this seldom happens. Our main interest is the characterization of pathways involved in the trait and we know genes are multifunctional^29^. What is relevant is that the independence of seed transcripts leads to quite different modules. In Fig. 2B, we show pairwise Jaccard coefficients between modules within each brain region-specific network. For all brain regions, the overlap between modules is practically zero, therefore the modules hold different transcripts (see supplementary table S5). Extreme brain regions regarding modules composition overlap are the age-TGCN in substantia nigra, whose modules show no overlap (Fig. 2C) and the age-TGCN in hippocampus, which showed a significant pairwise overlap (Fisher exact test *P* < 0.05) between three of its modules, *CLEC5*, *MARCO* and *VENTX* modules (Fig. 2D).

### Targeted GCNs provide more specific modules than WGCNA

WGCNA is the most widely extended hypothesis free approach to create genome wide gene co-expression networks. Since TGCNs are focused on a specific trait and its transcript pool is restricted to gene expression pathways associated with the trait, TGCNs are smaller and much more specific than hypothesis free GCNs which act on the whole transcriptome. For example, on average, the GTEx WGCNA networks we created for the 13 brain tissues use transcript pools of 15,281 genes in size and have nine modules average each network, with a mean size for each module of 1787 transcripts. The corresponding age-TGCNs use, on average, 426 transcripts in total and have 12 modules average each network with a mean module size of 37 transcripts. 35.9-fold more transcripts in WGCNA GCNs (see supplementary tables S6 and S7). Remarkably, even though TGCN modules are smaller in transcripts, TGCN achieves similar or higher functional abundance across all 13 brain regions, on average 67.95 and 44.02 functional abundance per module in TGCN and WGCNA respectively.

### Seed transcripts in targeted GCNs are orthogonal to WGCNA hub transcripts

We evaluated the relevance of the GTEx age-targeted seeds transcripts into the corresponding WGCNA modules. We found those transcripts we consider as seeds in TGCNs play no particular relevant role in their WGCNA module, showing a modest module membership, with an average percentile of 14.51 (see supplementary table S8). A plausible explanation for this is that in this case, the trait, age at death, has a subtle association with the genome wide gene expression profile.

We also investigated the possible role of WGCNA hubs transcripts into the age-TGCNs in brain samples. For each WGCNA module, we selected the top 10 transcripts with highest adjacency values (hub transcripts) and checked the overlap with the TGCNs modules from the same brain region. We found that only a small proportion (18.75%) of WGCNA hub transcripts significantly overlapped with at least one TGCN module from the same brain region (see supplementary table S9).

### TGCN modules tend to be subsets of single WGCNA modules

In order to better understand the relation between WGCNA modules and TGCN modules, we investigated how transcripts in a TGCN spread across the WGCNA GCNs created from the same gene expression profile. We found that, across all GTEx brain regions, 75.95% age-TGCN modules are included within a single WGCNA module while 16.46% WGCNA modules contribute significantly to more than one age-TGCN.

See, for example, the crosstab plot (see “Methods”) comparing the WGCNA GCN (6 modules) and the age-TGCN within the caudate brain region (16 modules) (supplementary Fig. S2).

All the transcripts not included within the age-TGCN fell into the cells of the rightmost column and only the *MT1G, GDF15* and *IGF2BP2* modules significantly overlapped with more than one module while the rest of modules overlapped with just a single module. WGCNA modules which include any transcript appearing in the corresponding TGCN, show significant overlap with more than one TGCN module. See for example, the yellow module including entirely six modules out of the 16 TGCN total modules. At the same time, only 53.58% of the transcripts in that module are used in TGCN modules. This suggests that TGCN modules’ functions are local to single WGCNA modules and by virtue of using only those transcripts that contribute to predict the trait, and those that co-express with them, they filter out all functions not relevant to the trait.

### TGCNs show specificity to the trait and brain region

TGCNs are a set of transcript-based modules, led by seed transcripts with no association to any other seed through mRNA levels, and that also resemble independent pathways within each module. We hypothesize that the pathways play a biological role, mediated by gene expression, in relation to the targeted trait. Therefore, the *APP-*targeted GCN that models the role of APP in the AD phenotype is assumed to describe the pathways APP is involved with. To what extent, do those pathways emerge from the fact that we are predicting a trait that is really associated with the gene expression of the samples used? What would happen if instead of using *APP* as the target, we would use as target a totally irrelevant gene in the *omics plus trait* context involved? Would the TGCN approach be specific enough to detect no relevant signal when there is actually none? To shed some light about these questions, we conducted an experiment with six genes not previously related with AD. The selection of control genes has been based on two criteria: (1) the selected control genes must be well studied, and their function characterized, to minimize the chance of eventually finding them associated with AD; (2) they must be functional, in the sense that they are associated with diseases other than of neurodegenerative nature. In supplementary table S10, the disease associated with each control gene is available.

Firstly, within all 10 LASSO bootstraps for *APP*, the minimum test set R^2^ for the linear model using the seeds selected through that bootstrap is 0.935, mean of 0.952. In all 10 LASSO bootstraps for all the other six genes, the maximum R^2^ was 0.702 and the minimum was 0.149 (see supplementary table S10, supplementary Fig. S3A). Secondly, the seeds detected by the method in all ten LASSO bootstraps for *APP* were nine. In three of the six other TGCNs, the method detected no seeds in any of the runs. In the other three, the method detected only two seeds in each (supplementary table S10, supplementary Fig. S3B). Finally, the average functional abundance of modules in the *APP*-TGCN, measured as the sum of -log_10_(Pval) for all enriched terms, is 293.9. We see practically no average enrichment in the other six networks, with 10.8 enrichment on average for the best network, *TNNT2*-TGCN. This is a 26-fold difference (see supplementary table S10, supplementary Fig. S3C), which we consider as a strong indication of TGCN specificity.

### Seed transcripts and co-expression modules of the *APP*-TGCN replicate in two alternative AD cohorts

**Figure 3:**
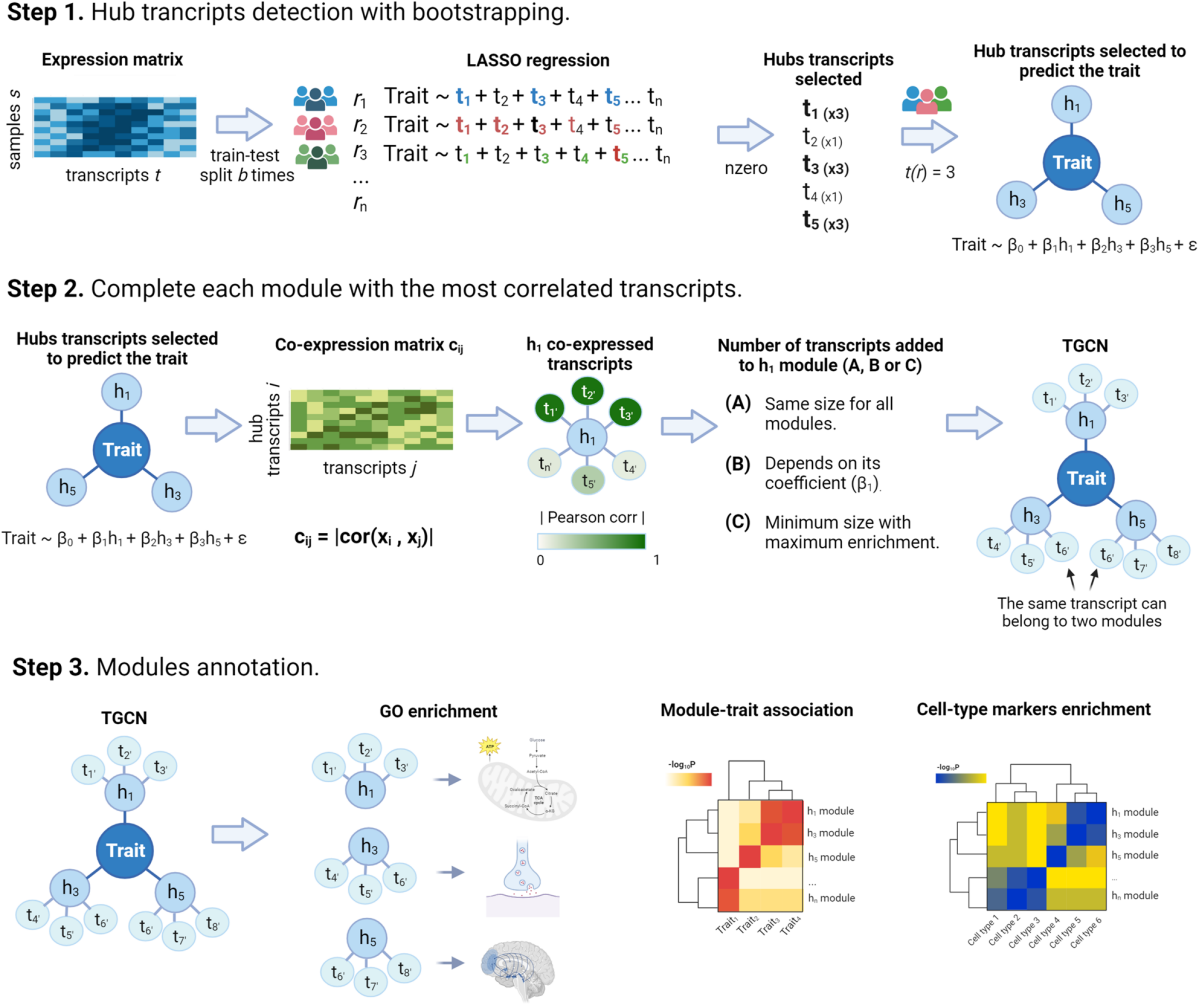
Workflow for the generation of targeted co-expression networks. The pipeline to create a TGCN is split into three steps (1) select the specific transcripts for the trait of interest (i.e. seed transcripts) based on a bootstrapped LASSO, (2) create the TGCN around those transcripts and (3) annotate the network modules. Step 1: to identify the transcripts that best predict the trait of interest, bootstrapped LASSO is used based on gene expression. Bootstrapped LASSO increases reliability of selected transcripts (seeds): the LASSO model is created *b* times with different train and test resamples. After all bootstraps, the TGCN software reports transcripts selected one or more times, along with how many times they were selected. Seed transcripts are selected on the basis of their ratio of appearance across bootstraps, *r*. Those transcripts are then used within a linear regression model as predictors to evaluate their prediction power on the trait and, therefore, the relevance of gene expression predicting that trait. Step 2: each seed transcript is the seed for a co-expression module. Modules are completed by adding the genes more co-expressed with the seed based on the absolute *Pearson* correlation*.* To determine how many transcripts to add to each module, the algorithm offers three options. In the first option, all seed transcripts are equally relevant within the network, therefore all modules should have the same size (i.e. 100 transcripts). In the second option, the higher the magnitude of the coefficient in the linear model, the more relevant the seed is within the TGCN. Therefore, the seed transcript with the highest coefficient receives the maximum number of transcripts (i.e. 100 transcripts). In the third option, we look for the minimum size of the module that reports the best enrichment possible.

TGCNs are constructed in three steps, by (1) detecting the seeds with model bootstrapping, (2) constructing the co-expression modules from those seeds and (3) annotating them (see Fig. 3). We constructed an *APP*-TGCN on the ROSMAP cohort (see materials) for *r* values of 8, 9 and 10. Thoroughly the paper, we focused on the ratio of appearance of eight, as it is a good trade-off between *APP* transcript expression prediction and complexity (i.e., it has 25 modules and 880 transcripts in total). We wanted to shed light about the *APP* transcript role in AD from brain samples gene expression. To assess whether this TGCN replicates, we used two alternative cohorts, Mayo and MSBB (see “Materials”). The seeds detected in ROSMAP with a minimum ratio of appearance of eight lead to a linear model with high *APP* predictivity (R^2^ = 0.97). These seeds also showed a high *APP*-predictivity in Mayo and MSBB cohorts, with R^2^ of 0.82 and 0.86, respectively (see supplementary table S11 for details on all models for *r* values of 8, 9 and 10). To properly assess whether those values of R^2^ were better than random chance, a permutation analysis was applied. To this end, we created 100,000 *APP*-predicting linear models with randomly chosen seed transcripts (same size of relevant seeds) and compared their performance with the *APP*-predictive model created with the relevant seed transcripts. This procedure was applied separately for both Mayo and MSBB. Results showed that the *APP*-predictive model created with the relevant transcripts performs better than *APP*-predictive models created with randomly chosen seed transcripts, leading to empirical p-values of statistical significance *P* < 0.059 and *P* < 4 × 10^–5^, respectively (see supplementary table S11, supplementary Fig. S4A,B). This suggests that seed transcripts discovered in ROSMAP reasonably explain *APP* transcript expression at the other two cohorts as well, nominally in Mayo and clearly in MSBB. Additionally, we evaluated how critical it would be to not find some of the seed transcripts in the expression matrix of the replication set. To this end, a permutation test was applied to compare the performance of the model created with the real seeds with the models created (1) removing each seed individually; (2) removing sets of five seeds (10,000 simulations); (3) removing sets of 10 seeds (10,000 simulations). This strategy was applied for Mayo and MSSB cohorts separately. We observed that, since it is an additive model, the effects of missing seeds are not noticeable (see supplementary table S12).

In order to assess whether co-expression modules are also present in the replication cohorts, for each *APP*-TGCN module built on the ROSMAP dataset, we tested for significant overlaps in the modules of Mayo and MSBB *APP*-TGCNs (see supplementary Fig. S4C,D). Out of the 25 modules identified in ROSMAP, seven modules did not show a significant overlap with any other module of the replication networks. The small size of the unreplicated co-expression modules (average size is 40) might explain, in part, their low replicability. Regardless, the replicability of 18 out of 25 modules across three brain transcriptomic AD datasets, demonstrated the robustness of this new method.

### Insights into the biology of APP and its role in AD

The TGCN R software package generates, for each targeted network, a whole bundle of information, in HTML format, on the TGCN construction process and results annotation (see the TGCN GitHub for the one generated for *APP*-TGCN). All this is summarized in a table with an entry for each module (see supplementary table S13). The complete *APP* centric network is composed of 880 transcripts, spread across 25 modules, whose size varies in the [10, 80] genes range (see “Methods”). The 25 seeds alone can explain most of the *APP* gene expression variation in a linear model (R^2^ 0.96). The CellTypeEnriched column of supplementary table S13 shows four modules that clearly contain cell marker enrichment, including the *ATP1B1* module enriched for dopaminergic neuron markers (Fisher’s exact *P* < 4.55^.^10^–3^), the *ABCD3* module, enriched for astrocytic markers (*P* < 1.083^.^10^–3^), the *FRZB* module enriched for mural cell markers (7.761^.^10^–4^), and the *NFASC* module enriched for oligodendrocyte markers (3.899^.^10^–4^). This adds evidence to the already known complex expression profile of *APP* in AD brains and different expression patterns across brain cell types. After multiple test corrections, the eigengenes of two of these modules were significantly correlated with AD status, i.e., the *ATP1B1* and *NFASC* modules (*P* < 0.038 y *P* < 7.02^.^10^–4^, respectively). The *ATP1B1* module is enriched for GO biological processes like synaptic vesicle cycle (GO:0,099,504, *P* < 3.65^.^10^–4^) and vesicle mediated transport in synapse (GO:0,099,003, *P* < 4.46^.^10^–4^). The *NFASC* module seems to be involved in the development of neurons, particularly through axons in the neurons since this module is enriched for terms like main axon (GO:0,044,304, *P* < 9.32^.^10^–5^) and axon development (GO:0,061,564, *P* < 6.49^.^10^–4^). Both *ATP1B1* and *NFASC* modules replicated in two independent cohorts. Specifically, *AT1BP1* module replicated in Mayo and MSBB cohorts with empirical p-values of *P* < 2.76^.^10^–9^ and *P* < 1.96^.^10^–10^, respectively. *NFASC* module replicated in Mayo and MSBB cohorts, reporting *P* < 4.51^.^10^–4^ and *P* < 1.03^.^10^–11^, respectively.

Additionally, the *HNRNPA2B1* module, showed a strong association with AD case/control status (*P* < 2.13^.^10^–5^), and seems to be involved in the metabolism of RNA through GO BP terms like RNA metabolic process (GO:0,016,070, *P* < 1.23^.^10^–10^), gene expression (GO:0,010,467, *P* < 1.73^.^10^–8^), particularly seems to be specialized in splicing, through the reactome term mRNA splicing (REAC:RHSA-72163, *P* < 6.89^.^10^–4^). This module also replicated in Mayo and MSBB cohorts (*P* < 3.16^.^10^–4^ and *P* < 1.83^.^10^–15^, respectively).

## Discussion

We have developed a new hypothesis-driven co-expression modeling approach as an alternative or to complement WGCNA-like networks (see Fig. 1), targeted co-expression networks (TGCNs). The approach is divided into three main steps: (1) seed transcript detection, (2) co-expression modules creation and (3) network annotation. The first step of the pipeline uses bootstrapped LASSO in order to select the most relevant transcripts (seed transcripts) in predicting the target trait. In this high dimensional data scenario, transcripts selected by LASSO can be slightly different from run to run. To address this sign of instability, we bootstrap the LASSO. Previous studies have demonstrated that bootstrapped LASSO increases the stability of the features selected^30,31^. As a proof of concept, we used LASSO to predict the age of death of the donors using transcriptomics from 13 different brain regions from the GTEx project^32^. Previous studies also identified a set of age-related genes for each tissue in the GTEx cohort. Yang et al*.*^33^ used a linear regression approach with 100 bootstrapping resamples to detect age-related genes within each tissue with at least 80 samples in size from an early version of GTEx (it did not include any brain tissue as all were smaller than 80 samples). Wang et al.^34^ ranked the age-related genes for the same 13 brain regions based on the Pearson correlation. For each brain region, the top 50 age-related genes for that region were used to create a predictive model. These models reported a mean RMSE of 7.89 ± 1.23, which is very close to the mean RMSE obtained with our models 7.42 ± 1.47 but we only require 12 transcripts on average to achieve that error (see supplementary table S2).

The second step of the TGCN approach characterizes the relation between the trait and the transcriptomic profiling. This is precisely what we have pursued by creating an *APP*-TGCN on AD samples: to shed light on the role of the *APP* gene within AD. Other works approach the AD phenotype from transcriptomics by extracting the most relevant features in predicting AD. LASSO, or LASSO combined with other approaches of feature selection (Boruta’s algorithm or differential expression analysis), have been applied for this task using a variety of data types, including MRI^35–37^, single nucleotide polymorphisms imputed from whole genome sequencing^38,39^, microarray^40,41^, plasma lipid levels^42^ and, of course transcriptomics^43,44^. However, since AD is a complex disease affected by multiple factors, we propose to approach the AD phenotype through a specific trait, as a way to simplify this challenging task, i.e. this is, precisely, the main motivation behind TGCNs. Naturally, the selected trait should be strongly associated with AD. In particular, we focused on the *APP* gene, which has a relevant role in AD^45^. Therefore, we created an *APP*-TGCN from the transcriptomics of the ROSMAP cohort. The model predicting the *APP* gene expression values reported a 0.97 R^2^. To the best of our knowledge, there is no other study where an *APP*-predictive model was created using transcriptomics data from human controls and AD donors. The reliability of the seed transcripts selected by TGCN was assessed in two independent cohorts, Mayo and MSBB, with R^2^ of 0.82 and 0.86, respectively. Amongst the modules within the *APP*-TGCN, the module led by the *HNRNPA2B1* seed transcript, a heterogeneous nuclear ribonucleoprotein from the A2B1 family, stands out from the rest because of its high correlation with donors neuro status (*P* < 2.13 × 10^–5^). Donev et al. demonstrated that hnRNP A1 is involved in alternative splicing of *APP* exons 7 and 8. Berson et al. found that both hnRNP proteins A1 and A2/B1 were depleted in entorhinal cortex samples from AD patients. In addition, HnRNP A2/B1 protein has also been linked to other neurodegenerative diseases such as amyotrophic lateral sclerosis^48,49^. Another module highly correlated with donor neuro status (*P* < 7.02 × 10^–4^) was the one led by neurofascin (*NFASC)* seed transcript. Neurofascin is a transmembrane protein that plays an essential role in nervous system development and node of Ranvier function^50^. In this same line, Xu et al., 2014 reported that *APP* aggregates at nodes of Ranvier in the myelinated central nervous system axons but not in the peripheral nervous system. Bai et al. demonstrated, in the mouse brain, that *APP* interacts with neurofascin. Brinkmalm et al. detected that the concentrations of the synaptic protein neurofascin were significantly lowered in the CSF of AD donors. Furthermore, *NFASC* gene mutations were previously associated with autosomal recessive ataxia with demyelinating neuropathy^54^ and was also proposed as a candidate autoantigen in multiple sclerosis^55^. Both modules, the ones led by *HNRNPA2B1* and *NFASC* seed transcripts, were further replicated in the two independent cohorts (Fisher exact test *P* < 0.05), demonstrating that our findings are reliable, and the networks are robust.

Finally, we compared the TGCN networks with WGCNA networks on the same GTEx transcriptomic profiles. Since WGCNA clusters the whole pool of transcripts into modules, the resulting clusters tend to be very large in size^56–58^ and more difficult to interpret. Since TGCNs are focused on a specific trait, these networks are more than 30 times smaller than WGCNA networks but surprisingly, equally rich in functional annotations (see “Results”). Besides, we observe that the most important WGCNA modules are actually an amalgamation of a few of the TGCN modules which clearly demonstrates that TGCNs are a simpler and more specific way to describe a trait of interest in terms of transcriptomics.

The targeted co-expression model we propose has some limitations in terms of applicability. Obviously, it is not directly applicable when there is no specific trait involved. However, transcriptomic cohorts created with no specific research hypothesis focused on a specific trait can yet be approached with this model. In such scenarios, we would create a TGCN for each trait separately used as a target. But considering that a biologically interesting TGCN has the potential to generate a large volume of information, having to manage multiple TGCNs at the same time makes the interpretation of all those models a hard task. As we are aware of this, the TGCN software provides all information in an HTML report that can easily be inspected and queried across the large volume of information delivered to ease all network interpretation tasks.

## Methods

### Data acquisition and preprocessing

The GTEx Portal is an ongoing effort to build a comprehensive public resource to study tissue-specific gene expression and regulation^32^. Based on our interest in neurodegeneration, we selected bulk RNA-seq data from different brain regions (N = 13, supplementary table S1) were included. For each brain region, TPM gene counts were downloaded and log_2_ (TPM + 1) transformed. The expression matrix was preprocessed as follows: (1) retention of protein coding genes with HGNC symbol and a minimum expression with biomaRt R package^59^; (2) quantile normalization and (3) Z-Score normalization.

Bulk RNA-seq data from brain of donors of AD cases and healthy controls was obtained for three different cohorts of the AMP-AD consortium: The Religious Orders Study and Memory and Aging Project (ROSMAP) Study, the Mayo RNA-seq Study (MayoRNAseq) and The Mount Sinai Brain Bank (MSBB) Study. We selected ROSMAP as the discovery dataset since it has been widely used as a research resource for investigators around the globe in more than 400 publications^60^. The expression matrix of each dataset was preprocessed as follows: (1) retention of protein coding genes with HGNC symbol and a minimum expression with biomaRt R package; (2) quantile normalization; (3) batch effect correction using ComBat function from the SVA R package^61^; (4) correction by RNA integrity number, post-mortem interval, age of death and sex with the svaseq function from the SVA R package.

The ROSMAP cohort consists of 640 dorsolateral prefrontal cortex samples of individuals from groups of the religious orders from across the United States and lay persons around the Chicago metropolitan area (ROSMAP)^60,62^ (syn3219045). Participants were classified as sporadic late onset AD, elderly control, or mild cognitive impairment based on clinical and neuropathological exams. ROSMAP cohort demographics are represented in supplementary Fig. S5. Only participants classified as healthy controls and AD were kept for downstream analysis. ROSMAP bulk RNA-seq data was downloaded as FPKM counts together with the corresponding metadata and phenotype. The same data preprocessing as ROSMAP was applied.

The Mayo cohort includes whole transcriptome data for 276 temporal cortex samples from individuals with caucasian ancestry with neuropathological diagnosis of AD, progressive supranuclear palsy, pathologic aging or elderly controls without neurodegenerative diseases^63^ (syn5550404). Normalized gene counts from the temporal cortex were downloaded together with donor phenotype. Samples that did not pass quality control were removed, that is, the subjects with low % mapped reads (< 85%) or sex discrepant gene counts and samples with gene body coverage ratio of > 3.0. For more information about the QC, please see (syn20818651). Then, the same data preprocessing as ROSMAP was applied.

MSBB brain data were obtained from the Mount Sinai/JJ Peters VA Medical Center Brain Bank (syn3159438). The dataset includes 214 frontopolar prefrontal cortex samples. Only participants classified as healthy controls (CDR = 0) and AD donors (CDR > 3) were kept for downstream analysis. RNA-seq raw counts were downloaded from synapse and transformed to RPKM. Then, the same data preprocessing as ROSMAP was applied.

### TGCN workflow

We have divided the generation of TGCN networks in three steps (Fig. 1). First, we select the specific transcripts for the trait of interest (i.e. seed transcripts) with LASSO regression^27^. Then, we create the network around those transcripts and annotate the modules.

#### Seed transcripts identification

Using the transcriptomic data as input and the trait of interest as outcome, we apply LASSO regression to identify the more relevant transcripts (Fig. 3, step 1). LASSO is one of the most popular regularization methods for linear models. It is characterized by the inclusion of a L_1_ penalty function at its cost function. The penalty is the sum of the absolute value of the $\beta$ coefficients at the linear model compound by p predictors multiplied by $\lambda$ (see below). The LASSO cost function is as follows:

$${R}_{\lambda }=RSS+\lambda {\sum }_{j=1}^{p}|{\beta }_{j}|,$$

where RSS (Residual Sum of Squares) is the sum of quadratic errors when predicting the dependent variable with each training sample. This penalty term will zero out some coefficients, giving the LASSO regression the capability of feature selection^64^, required to identify the seeds. This property is optimized via the $\lambda$ hyperparameter, estimated with cross-validation^65^. Gene expression profiles are very particular datasets for machine learning: they are highly dimensional (the number of potential predictors is of the order of thousands) and the predictors show high collinearity (groups of genes present high correlation amongst them). In these situations, it is easy to find many alternative linear models with approximately similar predictive performance. This leads to algorithm instability, when a small change in the training samples imply an appreciable change in the gene set used at the final LASSO model. In order to increase LASSO stability, we bootstrap the LASSO. Increasing stability also improves the reliability of the predictors (transcripts in this case) selected as relevant by LASSO, i.e., the LASSO solution. To this end, we generate *b* different LASSO solutions from *b* bootstrap train-test resamples. Each LASSO model in the bootstrap is created using a 5-folds cross-validation with the cv.glmnet function from glmnet package^66^ and only those transcripts with non-zero coefficients are considered.

Once all *b* LASSO models are created, the transcripts selected by the LASSO are grouped based on the number of times the transcript was selected, *r*, across the *b* runs, with $1\le r\le b$. If the user does not specify a value for *r*, then for each value of *r*, the software creates a linear model to predict the trait of interest using as predictors the transcripts selected *r* or more times. Models are created with the Caret R package^67^ with a 5-folds cross-validation repeated *b* times (i.e., *b* is 10 by default). It is up to the user to find the right trade-off between complexity of the model and variance of the trait explained by it (more complex models lead to better approximation of the trait, but they are harder to manage as they will be larger). The TGCN software assists in this process through the generation of a detailed report of the whole analysis (see the front page of the software at GitHub for a detailed explanation).

#### Building co-expressed modules around each seed transcript

After selecting the seeds, the TGCN software characterizes the pathways involved to better describe how the seeds are associated with the trait. To do this, we select the most co-expressed transcripts for each seed (Fig. 3, step 2). We define co-expression of a pair of transcripts as the absolute value of their Pearson correlation.

To optimize on the number of transcripts/features to include in a module, the TGCN R Package offers three different approaches. The simplest approach assumes that all seed transcripts are equally relevant, therefore, all modules are created with the same number of transcripts, i.e., those with highest correlation with the seed (100 by default). An alternative option assumes that seeds have different relevance, represented by their coefficient, in absolute value, at the linear regression model created to predict the trait. Thus, the resulting module size is proportional to the relevance, with a minimum size specified as a parameter (10 by default). The seed transcript with the highest coefficient will lead to the largest module (100 transcripts by default), followed in size by the second most relevant seed transcript and so on. The size of module *n*, S_n_ is.

$${S}_{n}={S}_{1}\cdot \frac{{\beta }_{n}}{{\beta }_{1}},$$

where S_1_ is the size of the largest module, and ${\beta }_{1}$ and ${\beta }_{n}$ are the coefficients in the linear models that multiply at the seeds for the largest module and the *n* module, respectively. In the third approach, the one we have used thoroughly in the paper, for each seed transcript, we look for the minimum module size that reports the best enrichment possible estimated with the functional abundance (see the term definition below). For each seed, we start with (1) the list of all transcripts, ranked by their correlation with the seed (in absolute value) and (2) an initial module including the 10 top transcripts in the rank. We keep adding 10 transcripts iteratively while all transcripts added still show significant correlation with the seed after 5% BH^68^ multiple testing correction and the relative enrichment of the new module improves the older enrichment.

#### Seed transcripts characterization through module annotation

After all modules are created from their seed, the TGCN tool identifies which pathways are represented in them via comprehensive annotation (Fig. 3, step 3). Firstly, it identifies associations between the targeted modules and other traits of interest different from the target trait. To do this, we follow the same approach as WGCNA, i.e., we assess the association of the trait with the eigengene of each module (i.e. the first principal component of the expression profiles of its transcripts). The association is detected through the corWithCatTraits function from the CoExpNets R package, incorporated into the TGCN software. Secondly, TGCN tests whether the modules are cell type-specific by checking whether they are enriched with cell-type markers using the genAnnotationCellType function from the CoExpNets R package^21,69^. Finally, it performs a functional enrichment analysis per module with the gost function from gprofiler2 R package^70^ using GO, KEGG, REAC and HP databases.

### Assessing the functional abundance of a geneset

The TGCN software needs to assess the biological relevance of genesets, i.e. seeds and modules within TGCNs. For such a purpose, we defined a simple measure based on both abundance and significance level of the terms extracted from a GSE (Gene Set Enrichment) analysis on the corresponding geneset. Given a geneset *G*, we carry out a functional enrichment analysis and obtain the enrichment result (i.e., a list of significantly enriched terms, along with their p-values) using the gost function from the gprofiler2 R package. The statistics is named *functional abundance* and is obtained by means of the expression:

$$\frac{1}{|G|} {\sum }_{i=1}^{n}-log10(pval({t}_{i}))$$

where pval(t_i_) is the enrichment p-value for term t_i_, n is the number of significant terms and |G| is the number of genes.

### Assessing independence of seed transcripts

As described above, when looking for the seed transcripts, the TGCN tool repeats LASSO *b* times with random train-test partitions. In each iteration, the mutual independence of the relevant seed transcripts is critical to, consequently, create modules that are independent and contain non-redundant biological information across them. To assess the potential level of association between pairs of seed transcripts selected for each value of the *r* variable (the ratio of appearance), we have performed two complementary experiments. The first one is a permutation test approach. For each LASSO iteration, we compare the maximum correlation across all possible pairs of seeds and the maximum correlation across the same number of pairs of random transcripts, not including the seeds. To assess the statistical significance of the comparison outcome we use a t-test. In the second approach, we assume that unrelated transcripts would yield very low functional abundance. Therefore, we measure the seeds' functional abundance as a geneset.

### Seed transcripts replication in independent cohorts

For the GTEx dataset, we tested the replicability of the age seeds detected in a specific brain region in each of the other 12 brain regions. To properly calibrate their effectiveness, we designed a permutation-based test where we compare the observed performance of the model created with the real seeds with the performance of 10,000 models created with randomly selected seeds of the same size. An empirical p-value of the test shows how probable it is to get a random model with better predictive capacity on age than the selected by our software. We assume the seeds are replicated in the other brain region when *P* < 0.05. This same permutation-based approach was used to test the replicability of *APP* seeds detected in ROSMAP in the two independent cohorts, Mayo and MSBB. Finally, to evaluate how critical it would be to not find some of the *APP* seed transcripts in the expression matrix of the replication set, a permutation test was applied to compare the performance of the model created with the real seeds with the models created (1) removing each seed individually; (2) removing sets of five seeds (10,000 simulations); (3) removing sets of 10 seeds (10,000 simulations). This strategy was applied for Mayo and MSSB cohorts separately.

### Creation of WGCNA networks

We compared the target GCNs created with our software with the networks created with WGCNA to demonstrate that (1) our modules are more specific than WGCNA ones since they were constructed around a trait, therefore, we can disentangle between the annotations specific to one trait or another (2) our networks are smaller and, therefore, more manageable; (3) our modules have comparable enrichment to WGCNA ones. A WGCNA network for the transcriptomic profiling of each brain region from the GTEx project was created (13 brain regions in total). To this end, the getDownstreamNetwork function from the CoExpNets R package^21^ was used. First, it creates a TOM (Topological Overlap Matrix) matrix and the corresponding dendrogram through the getAndPlotNetworkLong function with the following parameters: (1) beta = − 1 (if beta is < 0 then it is obtained automatically); (2) net.type = “signed”; (3) cor.type = “Pearson”; (4) mak.k.cutoff = 150 (connectivity parameter); (5) r.sq.cutoff = 0.8 (only beta values above this value for the adjusted linear regression model are considered). Then, this function applies a dynamic cutree algorithm to identify the modules through the cutreeDynamic function from the dynamicTreeCut R package with deep.split = 2. Finally, the output of the conventional WGCNA clustering is used as subsequent input to a k-means clustering algorithm for further refinement through the applyFastKMeans function. Specifically, 20 iterations of the k-means algorithm with a minimum exchange of 20 genes was applied. Finally, the relevance of each transcript in each module was calculated as in WGCNA, using the module membership (MM) as a metric, which represents the correlation between the transcript expression profile and the module eigengene. MM was calculated using the getMM function from the CoExpNets R package.

## Supplementary Information

Supplementary Information.

## Data Availability

GTEx bulk RNA-seq data (V8 release) is open data access that can be downloaded from the GTEx portal (https://gtexportal.org/home/). Bulk RNA-seq data from Alzheimer’s disease cases and healthy controls was obtained for three different cohorts from the AD Knowledge Portal (https://adknowledgeportal.org): ROSMAP study (10.7303/syn3219045), the Mayo RNA-seq Study (10.7303/syn5550404) and the MSBB Study (10.7303/syn3159438). Data from these studies is controlled access. Downloading data requires a Synapse account and the submission of a Data Use Certificate.

## Code availability

The TGCN R package is available on GitHub at this repository: https://github.com/aliciagp/TGCN.

## References

[1] Parikshak, N. N., Gandal, M. J. & Geschwind, D. H. Systems biology and gene networks in neurodevelopmental and neurodegenerative disorders. *Nat. Rev. Genet.* **16**, 441–458 (2015). 26149713 10.1038/nrg3934 PMC4699316

[2] Friedman, N., Linial, M., Nachman, I. & Pe’er, D. Using Bayesian networks to analyze expression data. *J. Comput. Biol. J. Comput. Mol. Cell Biol.* **7**, 601–620 (2000). 10.1089/106652700750050961 11108481

[3] Peng, J., Wang, P., Zhou, N. & Zhu, J. Partial correlation estimation by joint sparse regression models. *J. Am. Stat. Assoc.* **104**, 735–746 (2009). 19881892 10.1198/jasa.2009.0126 PMC2770199

[4] Margolin, A. A. *et al.* ARACNE: an algorithm for the reconstruction of gene regulatory networks in a mammalian cellular context. *BMC Bioinformatics* **7**(Suppl 1), S7 (2006). 10.1186/1471-2105-7-S1-S7 PMC1810318 16723010

[5] Faith, J. J. *et al.* Large-scale mapping and validation of Escherichia coli transcriptional regulation from a compendium of expression profiles. *PLoS Biol.* **5**, e8 (2007). 17214507 10.1371/journal.pbio.0050008 PMC1764438

[6] Meyer, P. E., Kontos, K., Lafitte, F. & Bontempi, G. Information-theoretic inference of large transcriptional regulatory networks. *EURASIP J. Bioinforma. Syst. Biol.* **2007**, 79879 (2007). 10.1155/2007/79879 PMC3171353 18354736

[7] Butte, A. J. & Kohane, I. S. Mutual information relevance networks: functional genomic clustering using pairwise entropy measurements. *Pac. Symp. Biocomput. Pac. Symp. Biocomput.* 418–429. 10.1142/9789814447331_0040 (2000). 10.1142/9789814447331_0040 10902190

[8] Steuer, R., Kurths, J., Daub, C. O., Weise, J. & Selbig, J. The mutual information: Detecting and evaluating dependencies between variables. *Bioinformatics* **18**, S231–S240 (2002). 12386007 10.1093/bioinformatics/18.suppl_2.s231

[9] Sobolevsky, S., Campari, R., Belyi, A. & Ratti, C. General optimization technique for high-quality community detection in complex networks. *Phys. Rev. E* **90**, 012811 (2014). 10.1103/PhysRevE.90.012811 25122346

[10] De Meo, P., Ferrara, E., Fiumara, G. & Provetti, A. Mixing local and global information for community detection in large networks. *J. Comput. Syst. Sci.* **80**, 72–87 (2014).

[11] Clauset, A., Newman, M. E. J. & Moore, C. Finding community structure in very large networks. *Phys. Rev. E* **70**, 066111 (2004). 10.1103/PhysRevE.70.066111 15697438

[12] Newman, M. E. J. Finding community structure in networks using the eigenvectors of matrices. *Phys. Rev. E* **74**, 036104 (2006). 10.1103/PhysRevE.74.036104 17025705

[13] Blondel, V. D., Guillaume, J.-L., Lambiotte, R. & Lefebvre, E. Fast unfolding of communities in large networks. *J. Stat. Mech. Theory Exp.* **2008**, P10008 (2008).

[14] Reichardt, J. & Bornholdt, S. Statistical mechanics of community detection. *Phys. Rev. E* **74**, 016110 (2006). 10.1103/PhysRevE.74.016110 16907154

[15] Mostafavi, S., Ray, D., Warde-Farley, D., Grouios, C. & Morris, Q. GeneMANIA: A real-time multiple association network integration algorithm for predicting gene function. *Genome Biol.* **9**(Suppl 1), S4 (2008). 10.1186/gb-2008-9-s1-s4 PMC2447538 18613948

[16] Langfelder, P. & Horvath, S. WGCNA: An R package for weighted correlation network analysis. *BMC Bioinformatics* **9**, 559 (2008). 19114008 10.1186/1471-2105-9-559 PMC2631488

[17] Gandal, M. J. *et al.* Broad transcriptomic dysregulation occurs across the cerebral cortex in ASD. *Nature* **611**, 532–539 (2022). 36323788 10.1038/s41586-022-05377-7 PMC9668748

[18] Ramaswami, G. *et al.* Integrative genomics identifies a convergent molecular subtype that links epigenomic with transcriptomic differences in autism. *Nat. Commun.* **11**, 4873 (2020). 32978376 10.1038/s41467-020-18526-1 PMC7519165

[19] Guelfi, S. *et al.* Regulatory sites for splicing in human basal ganglia are enriched for disease-relevant information. *Nat. Commun.* **11**, 1041 (2020). 32098967 10.1038/s41467-020-14483-x PMC7042265

[20] Forabosco, P. *et al.* Insights into TREM2 biology by network analysis of human brain gene expression data. *Neurobiol. Aging* **34**, 2699–2714 (2013). 23855984 10.1016/j.neurobiolaging.2013.05.001 PMC3988951

[21] Botía, J. A. *et al.* An additional k-means clustering step improves the biological features of WGCNA gene co-expression networks. *BMC Syst. Biol.* **11**, 47 (2017). 28403906 10.1186/s12918-017-0420-6 PMC5389000

[22] Molet, M., Stagner, J. P., Miller, H. C., Kosinski, T. & Zentall, T. R. Guilt by association and honor by association: The role of acquired equivalence. *Psychon. Bull. Rev.* **20**, 385–390 (2013). 23208768 10.3758/s13423-012-0346-3

[23] Ashburner, M. *et al.* Gene ontology: tool for the unification of biology. The Gene Ontology Consortium. *Nat. Genet.* **25**, 25–29 (2000). 10802651 10.1038/75556 PMC3037419

[24] Fabregat, A. *et al.* The reactome pathway knowledgebase. *Nucleic Acids Res.* **46**, D649–D655 (2018). 29145629 10.1093/nar/gkx1132 PMC5753187

[25] Kanehisa, M. & Goto, S. KEGG: Kyoto encyclopedia of genes and genomes. *Nucleic Acids Res.* **28**, 27–30 (2000). 10592173 10.1093/nar/28.1.27 PMC102409

[26] Köhler, S. *et al.* The human phenotype ontology in 2021. *Nucleic Acids Res.* **49**, D1207–D1217 (2021). 33264411 10.1093/nar/gkaa1043 PMC7778952

[27] Tibshirani, R. Regression Shrinkage and Selection via the Lasso. *J. R. Stat. Soc. Ser. B Methodol.* **58**, 267–288 (1996).

[28] Serrano-Pozo, A., Frosch, M. P., Masliah, E. & Hyman, B. T. Neuropathological alterations in Alzheimer Disease. *Cold Spring Harb. Perspect. Med.* **1**, a006189 (2011). 22229116 10.1101/cshperspect.a006189 PMC3234452

[29] Sánchez, J. A. *et al.* Modeling multifunctionality of genes with secondary gene co-expression networks in human brain provides novel disease insights. *Bioinformatics* **37**, 2905–2911 (2021). 33734320 10.1093/bioinformatics/btab175 PMC8479669

[30] Meinshausen, N. & Bühlmann, P. Stability selection. *J. R. Stat. Soc. Ser. B Stat. Methodol.* **72**, 417–473 (2010).

[31] Bach, F. R. Bolasso: model consistent Lasso estimation through the bootstrap. in *Proceedings of the 25th international conference on Machine learning* 33–40 (Association for Computing Machinery, New York, NY, USA, 2008). 10.1145/1390156.1390161.

[32] Lonsdale, J. *et al.* The genotype-tissue expression (GTEx) project. *Nat. Genet.* **45**, 580–585 (2013). 23715323 10.1038/ng.2653 PMC4010069

[33] Yang, J. *et al.* Synchronized age-related gene expression changes across multiple tissues in human and the link to complex diseases. *Sci. Rep.* **5**, 15145 (2015). 26477495 10.1038/srep15145 PMC4609956

[34] Wang, F. *et al.* Improved human age prediction by using gene expression profiles from multiple tissues. *Front. Genet.* **11**, (2020). 10.3389/fgene.2020.01025 PMC7546819 33101366

[35] Cui, X. *et al.* Adaptive LASSO logistic regression based on particle swarm optimization for Alzheimer’s disease early diagnosis. *Chemom. Intell. Lab. Syst.* **215**, 104316 (2021).

[36] Sun, Z., Fan, Y., M.d, B. P. F. L. & Giessen, M. van de. Detection of Alzheimer’s disease using group lasso SVM-based region selection. In *Medical Imaging 2015: Computer-Aided Diagnosis* vol. 9414 285–291 (SPIE, 2015).

[37] Lee, S. H., Yu, D., Bachman, A. H., Lim, J. & Ardekani, B. A. Application of fused lasso logistic regression to the study of corpus callosum thickness in early Alzheimer’s disease. *J. Neurosci. Methods* **221**, 78–84 (2014). 24121089 10.1016/j.jneumeth.2013.09.017 PMC4314964

[38] Yang, T. *et al.* Detecting genetic risk factors for Alzheimer’s disease in whole genome sequence data via Lasso screening. In *2015 IEEE 12th International Symposium on Biomedical Imaging (ISBI)* 985–989. 10.1109/ISBI.2015.7164036 (2015). 10.1109/ISBI.2015.7164036 PMC4578226 26413209

[39] Dondelinger, F., Mukherjee, S., & The Alzheimer’s Disease Neuroimaging Initiative. The joint lasso: High-dimensional regression for group structured data. *Biostatistics* **21**, 219–235 (2020). 10.1093/biostatistics/kxy035 PMC7868060 30192903

[40] Sharma, A. & Dey, P. A machine learning approach to unmask novel gene signatures and prediction of Alzheimer’s disease within different brain regions. *Genomics* **113**, 1778–1789 (2021). 33878365 10.1016/j.ygeno.2021.04.028

[41] Yu, W., Yu, W., Yang, Y. & Lü, Y. Exploring the key genes and identification of potential diagnosis biomarkers in Alzheimer’s disease using bioinformatics analysis. *Front. Aging Neurosci.* **13**, (2021). 10.3389/fnagi.2021.602781 PMC8236887 34194312

[42] Ma, Y.-H. *et al.* A panel of blood lipids associated with cognitive performance, brain atrophy, and Alzheimer’s diagnosis: A longitudinal study of elders without dementia. *Alzheimers Dement. Diagn. Assess. Dis. Monit.* **12**, e12041 (2020). 10.1002/dad2.12041 PMC7507431 32995461

[43] Abdullah, M. N., Wah, Y. B., Abdul Majeed, A. B., Zakaria, Y. & Shaadan, N. Identification of blood-based transcriptomics biomarkers for Alzheimer’s disease using statistical and machine learning classifier. *Inform. Med. Unlocked* **33**, 101083 (2022).

[44] Alamro, H. *et al.* Exploiting machine learning models to identify novel Alzheimer’s disease biomarkers and potential targets. *Sci. Rep.* **13**, 4979 (2023). 36973386 10.1038/s41598-023-30904-5 PMC10043000

[45] Zetterberg, H., Blennow, K. & Hanse, E. Amyloid β and APP as biomarkers for Alzheimer’s disease. *Exp. Gerontol.* **45**, 23–29 (2010). 19698775 10.1016/j.exger.2009.08.002

[46] Donev, R., Newall, A., Thome, J. & Sheer, D. A role for SC35 and hnRNPA1 in the determination of amyloid precursor protein isoforms. *Mol. Psychiatry* **12**, 681–690 (2007). 17353911 10.1038/sj.mp.4001971 PMC2684093

[47] Berson, A. *et al.* Cholinergic-associated loss of hnRNP-A/B in Alzheimer’s disease impairs cortical splicing and cognitive function in mice. *EMBO Mol. Med.* **4**, 730–742 (2012). 22628224 10.1002/emmm.201100995 PMC3494073

[48] Martinez, F. J. *et al.* Protein-RNA networks regulated by normal and ALS-associated mutant HNRNPA2B1 in the nervous system. *Neuron* **92**, 780–795 (2016). 27773581 10.1016/j.neuron.2016.09.050 PMC5123850

[49] Kim, H. J. *et al.* Mutations in prion-like domains in hnRNPA2B1 and hnRNPA1 cause multisystem proteinopathy and ALS. *Nature* **495**, 467–473 (2013). 23455423 10.1038/nature11922 PMC3756911

[50] Zonta, B. *et al.* Glial and neuronal isoforms of Neurofascin have distinct roles in the assembly of nodes of Ranvier in the central nervous system. *J. Cell Biol.* **181**, 1169–1177 (2008). 18573915 10.1083/jcb.200712154 PMC2442198

[51] Xu, D.-E. *et al.* Amyloid precursor protein at node of Ranvier modulates nodal formation. *Cell Adhes. Migr.* **8**, 396–403 (2014). 10.4161/cam.28802 PMC4594432 25482638

[52] Bai, Y. *et al.* The in vivo brain interactome of the amyloid precursor protein. *Mol. Cell. Proteomics MCP* **7**, 15–34 (2008). 17934213 10.1074/mcp.M700077-MCP200

[53] Brinkmalm, G. *et al.* A parallel reaction monitoring mass spectrometric method for analysis of potential CSF biomarkers for Alzheimer’s disease. *Proteomics Clin. Appl.* **12**, (2018). 10.1002/prca.201700131 29028155

[54] Monfrini, E. *et al.* Neurofascin (NFASC) gene mutation causes autosomal recessive ataxia with demyelinating neuropathy. *Parkinson. Relat. Disord.* **63**, 66–72 (2019). 10.1016/j.parkreldis.2019.02.045 30850329

[55] Mathey, E. K. *et al.* Neurofascin as a novel target for autoantibody-mediated axonal injury. *J. Exp. Med.* **204**, 2363–2372 (2007). 17846150 10.1084/jem.20071053 PMC2118456

[56] Zhai, X., Xue, Q., Liu, Q., Guo, Y. & Chen, Z. Colon cancer recurrence-associated genes revealed by WGCNA co-expression network analysis. *Mol. Med. Rep.* **16**, 6499–6505 (2017). 28901407 10.3892/mmr.2017.7412 PMC5865817

[57] Yin, X. *et al.* Identification of key modules and genes associated with breast cancer prognosis using WGCNA and ceRNA network analysis. *Aging* **13**, 2519–2538 (2020). 33318294 10.18632/aging.202285 PMC7880379

[58] Liu, X., Hu, A.-X., Zhao, J.-L. & Chen, F.-L. Identification of key gene modules in human osteosarcoma by co-expression analysis weighted gene co-expression network analysis (WGCNA). *J. Cell. Biochem.* **118**, 3953–3959 (2017). 28398605 10.1002/jcb.26050

[59] Durinck, S. *et al.* BioMart and Bioconductor: a powerful link between biological databases and microarray data analysis. *Bioinforma. Oxf. Engl.* **21**, 3439–3440 (2005). 10.1093/bioinformatics/bti525 16082012

[60] Bennett, D. A. *et al.* Religious orders study and rush memory and aging project. *J. Alzheimers Dis. JAD* **64**, S161–S189 (2018). 29865057 10.3233/JAD-179939 PMC6380522

[61] Leek, J. T., Johnson, W. E., Parker, H. S., Jaffe, A. E. & Storey, J. D. The sva package for removing batch effects and other unwanted variation in high-throughput experiments. *Bioinformatics* **28**, 882–883 (2012). 22257669 10.1093/bioinformatics/bts034 PMC3307112

[62] Bennett, D. A., Schneider, J. A., Arvanitakis, Z. & Wilson, R. S. Overview and findings from the religious orders study. *Curr. Alzheimer Res.* **9**, 628–645 (2012). 22471860 10.2174/156720512801322573 PMC3409291

[63] Allen, M. *et al.* Human whole genome genotype and transcriptome data for Alzheimer’s and other neurodegenerative diseases. *Sci. Data* **3**, 160089 (2016). 27727239 10.1038/sdata.2016.89 PMC5058336

[64] Muthukrishnan, R. & Rohini, R. LASSO: A feature selection technique in predictive modeling for machine learning. In *2016 IEEE International Conference on Advances in Computer Applications (ICACA)* 18–20. 10.1109/ICACA.2016.7887916 (2016).

[65] Browne, M. W. Cross-validation methods. *J. Math. Psychol.* **44**, 108–132 (2000). 10733860 10.1006/jmps.1999.1279

[66] Friedman, J. H., Hastie, T. & Tibshirani, R. Regularization paths for generalized linear models via coordinate descent. *J. Stat. Softw.* **33**, 1–22 (2010). 20808728 PMC2929880

[67] Kuhn, M. Building predictive models in R using the caret package. *J. Stat. Softw.* **28**, 1–26 (2008).27774042

[68] Benjamini, Y. & Hochberg, Y. Controlling the false discovery rate: A practical and powerful approach to multiple testing. *J. R. Stat. Soc. Ser. B Methodol.* **57**, 289–300 (1995).

[69] García-Ruiz, S. *et al.* CoExp: A web tool for the exploitation of co-expression networks. *Front. Genet.* **12**, 630187 (2021). 33719340 10.3389/fgene.2021.630187 PMC7943635

[70] Kolberg, L., Raudvere, U., Kuzmin, I., Vilo, J. & Peterson, H. gprofiler2—an R package for gene list functional enrichment analysis and namespace conversion toolset g:Profiler. *F1000Research* **9**, ELIXIR-709 (2020). 10.12688/f1000research.24956.1 PMC7859841 33564394

