# Author Correction: Targeted co-expression networks for the study of traits

**DOI:** 10.1038/s41598-024-79460-6

**Published:** 2024-11-18

**Authors:** A. Gómez‑Pascual, G. Rocamora‑Pérez, L. Ibanez, J. A. Botía

**Affiliations:** 1 Communications Engineering and Information Department, University of Murcia, 30100 Murcia, Spain; 2 Department of Genetics and Genomic Medicine Research and Teaching, UCL GOS Institute of Child Health, London, WC1N 1EH UK; 3 Department of Psychiatry, Washington University School of Medicine, Saint Louis, MO 63110 USA; 4 Department of Neurology, Washington University School of Medicine, Saint Louis, MO 63110 USA

Correction to: *Scientific Reports* 10.1038/s41598-024-67329-7, published online 19 July 2024

In the original version of this Article, the Funding section was incomplete.

“This work was supported by the Fundación Séneca-Agencia de Ciencia y Tecnología de la Región de Murcia (Spain) (21259/FPI/19) and the Spanish Council of Science and Innovation through the Grant PID2022-136306OB-I00.”

now reads

“This work was supported by the Fundación Séneca-Agencia de Ciencia y Tecnología de la Región de Murcia (Spain) (21259/FPI/19) and the Spanish Council of Science and Innovation through the Grant PID2022-136306OB-I00 funded by MICIU/AEI/10.13039/501100011033 and FEDER, UE.”

The original Article has been corrected.

