# Supplementary material for: Targeted co-expression networks for the study of traits
Source: Sci Rep. 2024 Jul 19;14:16675. doi: 10.1038/s41598-024-67329-7 (PMC11271532; doi:10.1038/s41598-024-67329-7)
Supplement: Supplementary file 1 — Supplementary Information. [file 41598_2024_67329_MOESM1_ESM.docx]

Targeted co-expression networks for the study of traits

A. Gómez-Pascual^1^, G. Rocamora-Pérez^2^, L. Ibanez^3,4^, J.A. Botía^1*^

^1^Communications engineering and information department, University of Murcia, 30100 Murcia, Spain

^2^Department of Genetics and Genomic Medicine Research & Teaching, UCL GOS Institute of Child Health, London WC1N 1EH, UK

^3^Department of Psychiatry, School of Medicine, Washington University, Saint Louis, MO 63130, US

^4^Department of Neurology, School of Medicine, Washington University in Saint Louis, MO 63130, US

*corresponding author ([juanbot@um.es](mailto:juanbot@um.es))

**
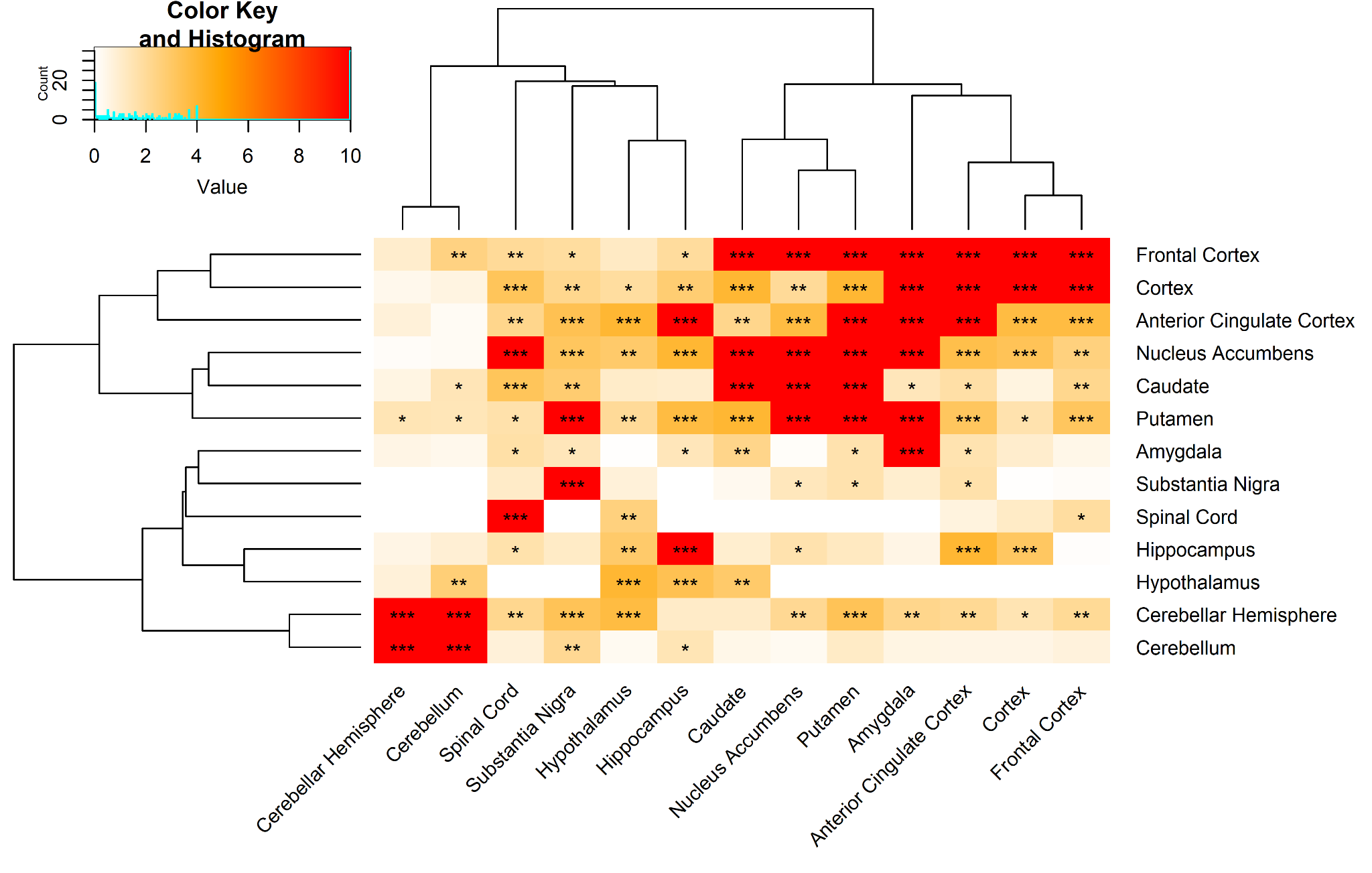
**

**Supplementary figure 1. Replication of age seed transcripts detected per brain region of the GTEx dataset in the other 12 brain regions.**

For the age seeds detected in a specific brain region, we tested their replicability in the other 12 brain regions. To this end, we applied a permutation test where the performance of the model created with the real seeds is compared with the performance of 10,000 models created with randomly selected seeds of the same size. In this heatmap, rows represent GTEx brain regions where the seed transcripts were extracted with bootstrapped LASSO while the columns represent the GTEx brain regions where the seed transcripts were tested. Each cell represents the -log_10_ of the p-value obtained from the permutation test of each pair of brain regions. *: *P*<0.05; **: *P*<0.01; ***: *P*<0.001.

**
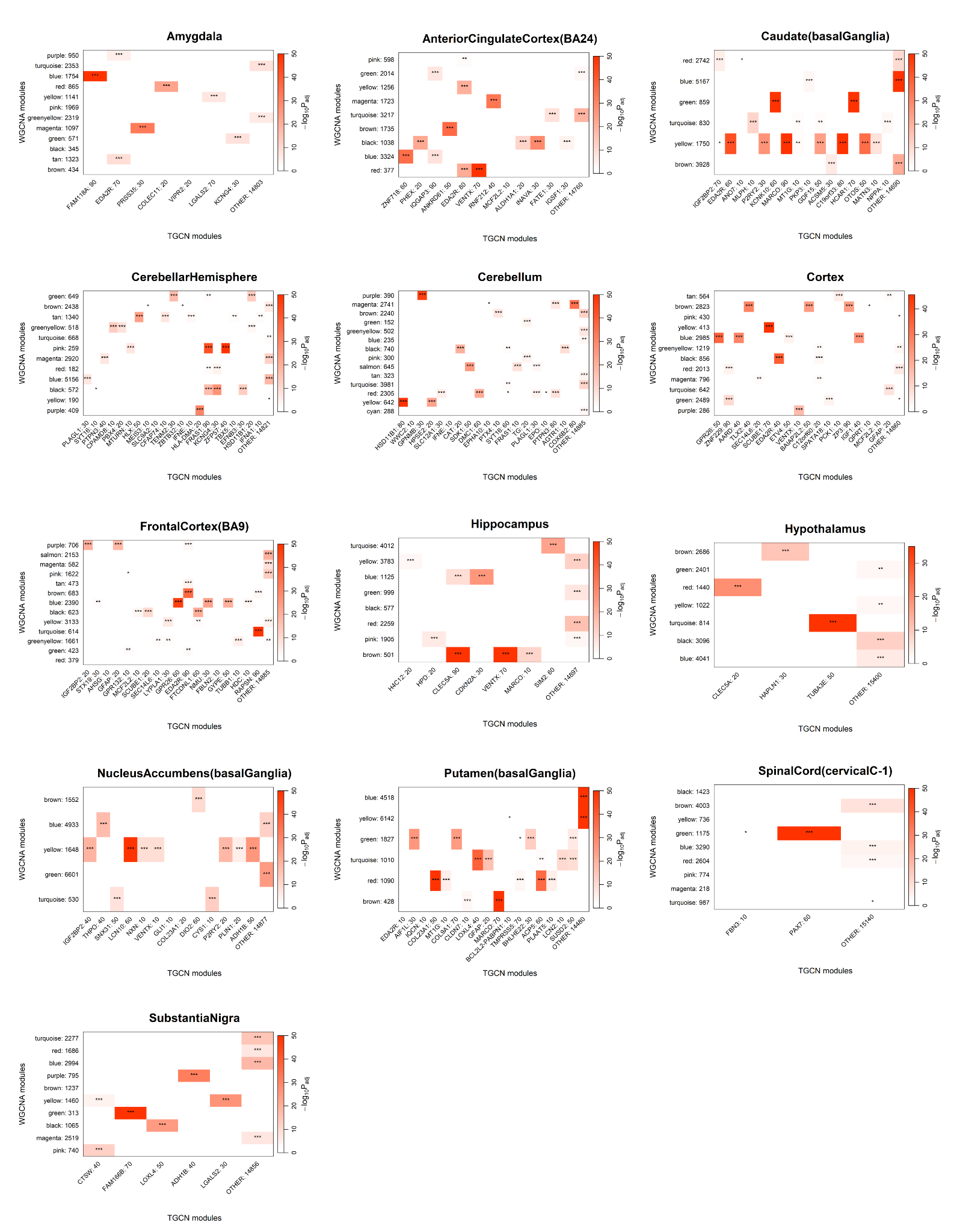
**

**Supplementary figure 2. Targeted modules tend to be subsets of single WGCNA modules.**

The overlap between WGCNA modules and TGCN modules in terms of transcripts composition is represented for all the GTEx brain regions. The color represents the -log_10_P of the overlap based on a Fisher Exact test. Only the significant overlaps are colored in the plot. We observe that WGCNA modules and our modules have a different structure.

**
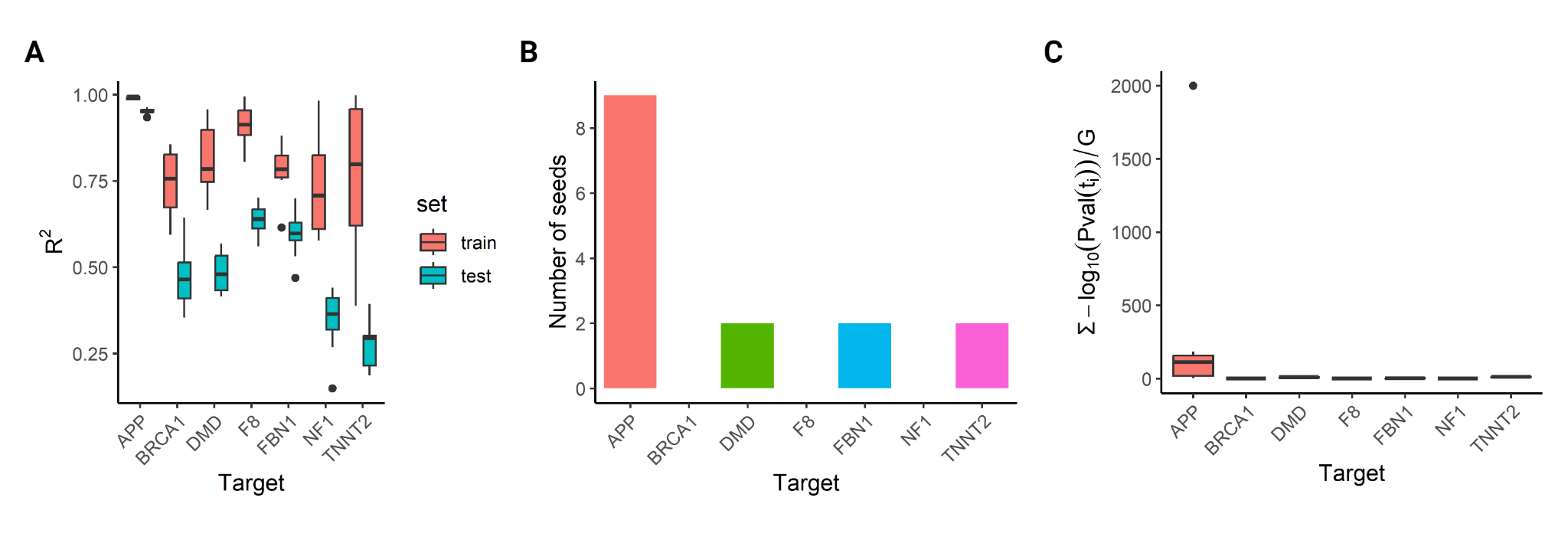
**

**Supplementary figure 3. TGCNs show specificity to the trait and brain region.**

**(A)** LASSO performance through 10 runs to predict *APP* and other six negative control transcripts using ROSMAP gene expression matrix as predictors.

**(B)** Number of seed transcripts selected in all 10 LASSO runs for *APP* and six negative control transcripts using ROSMAP gene expression matrix as predictors.

**(C)** The enrichment was estimated for each module of each transcript target GCN as the sum(-log_10_*P*).


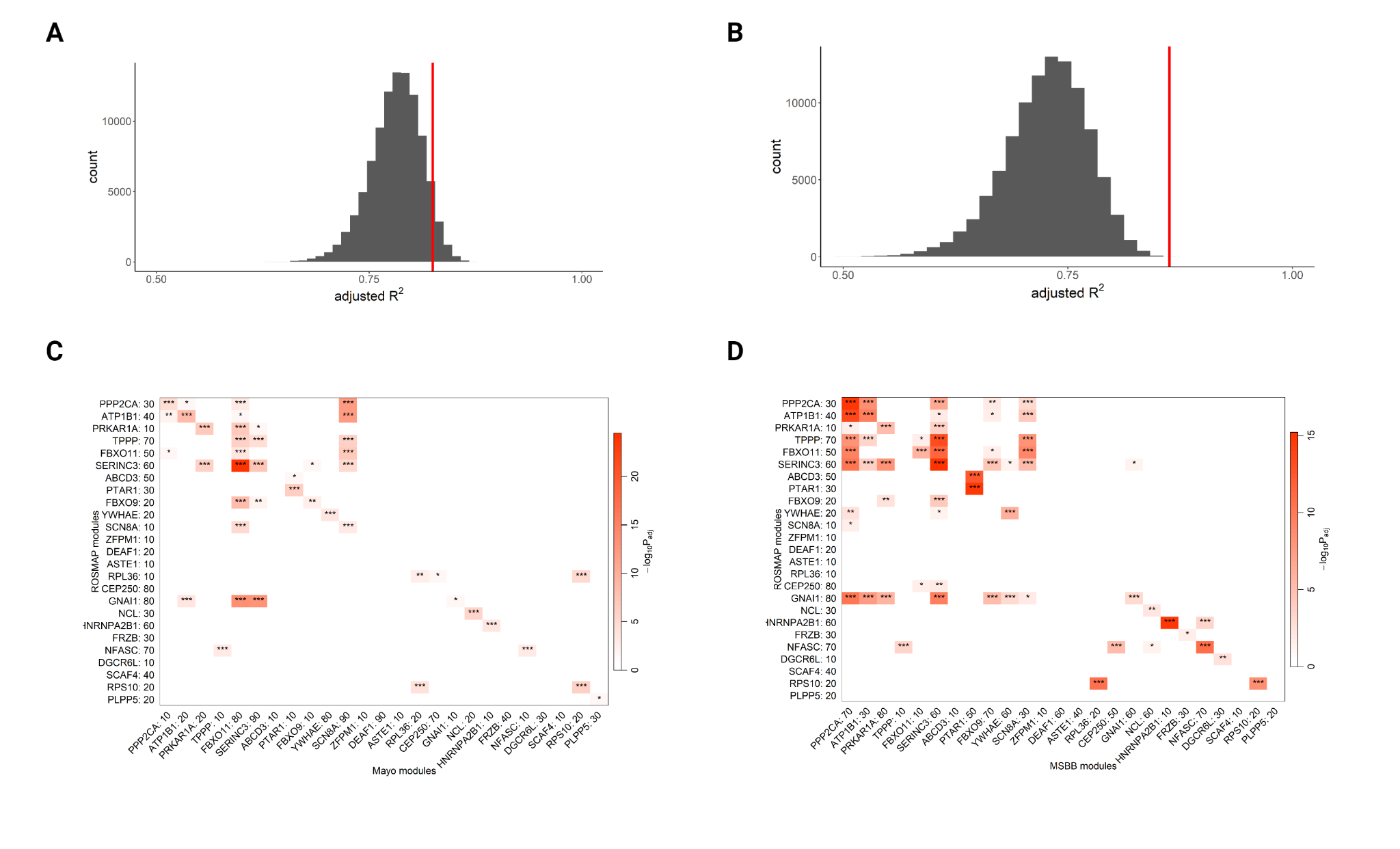


**Supplementary figure 4. *APP* seed transcripts and co-expression modules replicate in two alternative AD cohorts.** Seeds transcripts with a ratio of appearance of eight were selected for downstream analysis.

**(A, B)** Adjusted R^2^ distribution of 100,000 *APP*-predicting linear models with same size randomly chosen seed transcripts in Mayo (A) and MSBB (B) cohorts.

**(C, D)** Overlap between ROSMAP and Mayo and MSBB modules created around the same seed transcripts. Color scale corresponds to adjusted p-values from Fisher’s Exact test on the overlap. Only significant overlaps are colored.


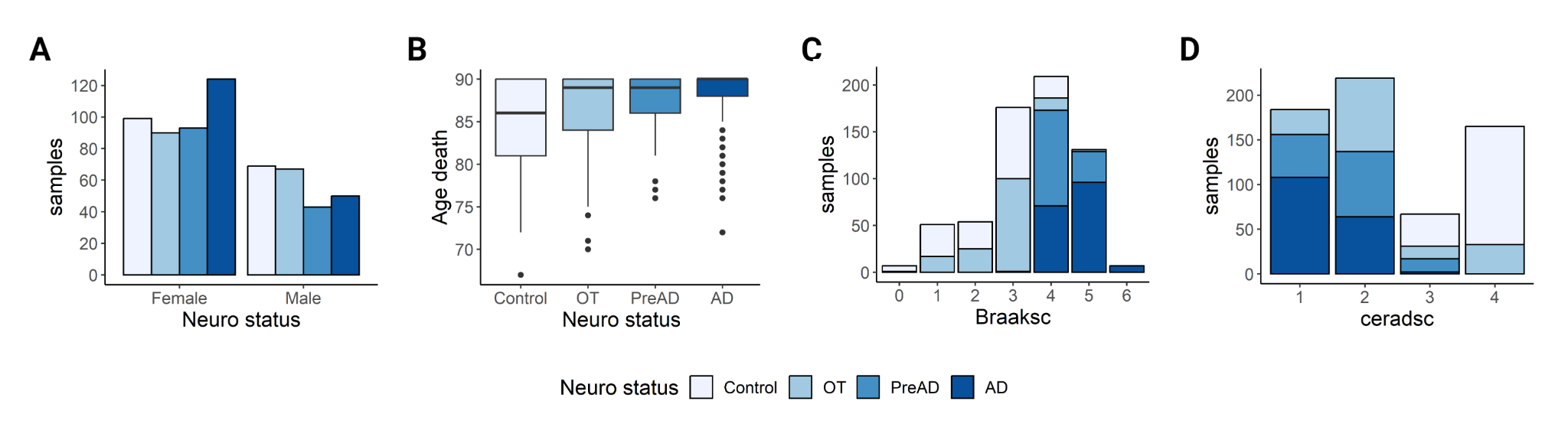


**Supplementary figure 5. ROSMAP cohort demographics.**

**(A)** Donors distribution per sex and neuro status. We observe a higher proportion of females compared to men.

**(B)** Age death distribution per neuro status. We observe a mean age of 87 years.

**(C)** Donors distribution per braaksc score and neuro status.

**(D)** Donors distribution per ceradsc score and neuro status.

OT, other; PreAD, Presymptomatic AD; AD, Alzheimer’s disease.

**Supplementary table 1.** **Main features of the datasets used in this study.** The number of transcripts represents the transcripts selected after filtering (for more details, see methods).

| **Purpose** | **Dataset** | **Brain tissue** | **Number of samples** | **Number of transcripts** | **Mean age of death** |
| --- | --- | --- | --- | --- | --- |
| Method characterization | GTEx (V8) | Amygdala | 152 | 15121 | 58 |
|  |  | Anterior cingulate cortex (BA24) | 176 | 15282 | 58 |
|  |  | Caudate (basal ganglia) | 246 | 15276 | 59 |
|  |  | Cerebellar Hemisphere | 215 | 15301 | 58 |
|  |  | Cerebellum | 241 | 15484 | 58 |
|  |  | Cortex | 255 | 15516 | 58 |
|  |  | Frontal Cortex (BA9) | 209 | 15442 | 59 |
|  |  | Hippocampus | 197 | 15161 | 59 |
|  |  | Hypothalamus | 202 | 15500 | 59 |
|  |  | Nucleus accumbens (basal ganglia) | 246 | 15264 | 59 |
|  |  | Putamen (basal ganglia) | 205 | 15015 | 58 |
|  |  | Spinal cord (cervical c-1) | 159 | 15210 | 59 |
|  |  | Substantia nigra | 139 | 15086 | 58 |
| Discovery | ROSMAP | Dorsolateral prefrontal cortex | 635 | 12545 | 87 |
| Replication | Mayo | Temporal cortex | 149 | 13396 | 82 |
|  | MSBB | Frontal pole | 261 | 13515 | 83 |

**Supplementary table 2. Statistics of LASSO models and seed transcripts model for each brain region of the GTEx dataset.** For each brain region, we applied LASSO regression with 10 different train-test partitions to predict the age of the participants using gene expression as predictors. These models, named LASSO models, reported a mean RMSE on test of 6.77 across the different brain regions and runs. Then, for each brain region, what we call *seed age transcripts* were defined as the transcripts selected by LASSO in at least 8/10 iterations. These seed transcripts were used as predictors in a linear regression model to predict the age of the participants. These models, called *seed transcripts models*, reported a mean RMSE on test of 5.93 across the different brain regions. For most of the regions, the models created with seed transcripts as predictors reported a lower RMSE on test compared to the LASSO models for the same brain region. The standard deviation represents the error of the 5-folds cross-validation (CV) repeated 10 times. Brain regions are ordered in descending order based on the test RMSE of the seed transcripts models.

|  | **LASSO models (10 runs)** | |  | **Seed transcripts models** | | | |
| --- | --- | --- | --- | --- | --- | --- | --- |
| **Brain region** | **Number of predictors** | **RMSE test**  **(10 runs mean ±SD)** |  | **Number of predictors** | **R^2^ CV (mean±SD)** | **RMSE CV (mean±SD)** | **RMSE test** |
| Frontal cortex (BA9) | 15442 | 7.322 ± 0.776 |  | 18 | 0.559 ± 0.126 | 6.240 ± 0.848 | 5.659 |
| Cerebellar hemisphere | 15301 | 7.063 ± 0.629 |  | 21 | 0.640 ± 0.165 | 5.514 ± 0.963 | 5.718 |
| Putamen (basal ganglia) | 15015 | 7.778 ± 0.713 |  | 17 | 0.612 ± 0.113 | 6.059 ± 0.840 | 5.964 |
| Anterior cingulate cortex (BA24) | 15282 | 8.121 ± 1.262 |  | 12 | 0.539 ± 0.156 | 6.867 ± 1.209 | 6.007 |
| Cerebellum | 15484 | 6.921 ± 0.404 |  | 19 | 0.732 ± 0.089 | 5.600 ± 0.601 | 6.178 |
| Cortex | 15516 | 7.237 ± 0.629 |  | 18 | 0.603 ± 0.122 | 6.396 ± 0.868 | 7.023 |
| Nucleus accumbens (basal ganglia) | 15264 | 8.418 ± 0.828 |  | 13 | 0.520 ± 0.148 | 6.643 ± 1.037 | 7.600 |
| Caudate (basal ganglia) | 15276 | 7.544 ± 0.900 |  | 16 | 0.593 ± 0.092 | 6.353 ± 0.746 | 7.646 |
| Hippocampus | 15161 | 8.504 ± 1.180 |  | 7 | 0.409 ± 0.135 | 8.372 ± 1.308 | 7.761 |
| Substantia nigra | 15086 | 9.557 ± 1.358 |  | 5 | 0.193 ± 0.155 | 10.040 ± 2.957 | 8.508 |
| Spinal cord (cervical C-1) | 15210 | 9.557 ± 1.185 |  | 2 | 0.266 ± 0.162 | 8.214 ± 1.605 | 9.006 |
| Amygdala | 15121 | 9.161 ± 1.614 |  | 7 | 0.413 ± 0.177 | 7.998 ± 1.732 | 9.644 |
| Hypothalamus | 15500 | 8.877 ± 1.160 |  | 3 | 0.263 ± 0.096 | 7.902 ± 1.035 | 9.690 |

**Supplementary table 3. Association between the number of times a transcript is selected by LASSO and its coefficient.** For each brain region of the GTEx dataset, we applied 10 LASSO runs to predict the age of death of the participants using as predictors the gene expression of the corresponding brain region. For each run, we extracted the transcripts selected by LASSO. We observed that the higher the number of times a transcript is selected by LASSO, the higher its coefficient in the LASSO models it was selected. To demonstrate this tendency, we created a linear regression model for each brain region, with the number of times selected as the response variable and the mean coefficient of the transcripts as the predictor. For all the brain regions, we observed a significant association between the number of times a transcript is selected and its mean coefficient (*P*<0.05). Figure 1B shows this relationship for cortex tissue.

| **Brain region** | **R^2^** | **adjusted R^2^** | **p-value** |
| --- | --- | --- | --- |
| Amygdala | 0.057 | 0.054 | 1.21^.^10^-05^ |
| Anterior Cingulate Cortex | 0.196 | 0.194 | 1.12^.^10^-24^ |
| Caudate | 0.175 | 0.173 | 9.09^.^10^-25^ |
| Cerebellar Hemisphere | 0.141 | 0.139 | 4.99^.^10^-16^ |
| Cerebellum | 0.146 | 0.144 | 7.65^.^10^-20^ |
| Cortex | 0.279 | 0.277 | 8.66^.^10^-38^ |
| Frontal Cortex | 0.141 | 0.140 | 2.36^.^10^-19^ |
| Hippocampus | 0.139 | 0.136 | 1.06^.^10^-10^ |
| Hypothalamus | 0.059 | 0.056 | 1.93^.^10^-05^ |
| Nucleus Accumbens | 0.159 | 0.158 | 1.65^.^10^-20^ |
| Putamen | 0.203 | 0.201 | 1.23^.^10^-23^ |
| Spinal Cord | 0.068 | 0.064 | 2.72^.^10^-05^ |
| Substantia Nigra | 0.126 | 0.123 | 3.41^.^10^-10^ |

**Supplementary table 4. Seed transcripts independence t-test results.** We applied 10 LASSO runs to predict the age of death of the donors using gene expression from cortex as predictors. For each run, we estimated the maximum correlation between the transcripts selected by LASSO and the maximum correlation between a set of random transcripts and compared the means using a t-test. This table shows the results of the figure 2A.

| **LASSO run** | **Maximum correlation between seed transcripts (95% CI)** | **Maximum correlation between random transcripts (95% CI)** | **t-test p-value** | **t-test mean diff** |
| --- | --- | --- | --- | --- |
| 1 | 0.486 (0.461-0.511) | 0.749 (0.712-0.787) | 1.28^.^10^-17^ | -0.263 |
| 2 | 0.499 (0.479-0.519) | 0.774 (0.749-0.800) | 1.078^.^10^-34^ | -0.275 |
| 3 | 0.493 (0.476-0.511) | 0.771 (0.750-0.793) | 1.311^.^10^-46^ | -0.278 |
| 4 | 0.498 (0.482-0.513) | 0.74 0(0.712-0.768) | 4.538^.^10^-28^ | -0.242 |
| 5 | 0.481 (0.462-0.500) | 0.743 (0.715-0.772) | 3.385^.^10^-29^ | -0.262 |
| 6 | 0.493 (0.464-0.523) | 0.738 (0.700-0.776) | 5.504^.^10^-16^ | -0.244 |
| 7 | 0.484 (0.462-0.506) | 0.748 (0.718-0.777) | 6.778^.^10^-27^ | -0.264 |
| 8 | 0.488 (0.457-0.519) | 0.753 (0.705-0.802) | 8.248^.^10^-12^ | -0.265 |
| 9 | 0.491 (0.462-0.519) | 0.769 (0.734-0.803) | 2.154^.^10^-19^ | -0.278 |
| 10 | 0.481 (0.462-0.500) | 0.775 (0.753-0.798) | 3.104^.^10^-42^ | -0.294 |

**Supplementary table 5. Targeted GCN modules show low or null overlap.** We created age-targeted GCNs for 13 different brain tissues from the GTEx dataset using the seed transcripts with a ratio of appearance of eight. For each tissue-specific GCN, we estimated the overlap between pairs of modules (except each module with itself) and reported the overlap with Jaccard index. The Jaccard index is 0 if the pairs of modules of the corresponding tissue-specific network don't share any transcripts and it is closer to 1 the more similar the pairs of modules are in terms of transcripts composition. Tissues are arranged in decreasing order of module overlap in terms of transcripts composition.

| **Brain region** | **Number of modules** | **Jaccard index (CI 95%)** |
| --- | --- | --- |
| Hypothalamus | 3 | 0 (0-0) |
| Spinal Cord | 2 | 0 (0-0) |
| Substantia Nigra | 5 | 0 (0-0) |
| Anterior Cingulate Cortex | 12 | 0.001 (0-0.002) |
| Cortex | 18 | 0.001 (0-0.002) |
| Frontal Cortex | 18 | 0.001 (0-0.002) |
| Cerebellar Hemisphere | 21 | 0.002 (0.001-0.003) |
| Nucleus Accumbens | 13 | 0.002 (0.001-0.004) |
| Putamen | 17 | 0.003 (0.001-0.004) |
| Cerebellum | 19 | 0.004 (0.002-0.005) |
| Caudate | 16 | 0.006 (0.002-0.009) |
| Amygdala | 7 | 0.015 (-0.006-0.036) |
| Hippocampus | 7 | 0.017 (0.004-0.031) |

**Supplementary table 6. GTEx WGCNA modules features per brain region.** Enrichment was calculated according to the methods section.

| **Brain region** | **TGCN size** | **Number of modules** | **Module size (mean, min, max)** | **Enrichment (mean, min, max)** |
| --- | --- | --- | --- | --- |
| Amygdala | 15121 | 12 | 1260 (345-2353) | 4.03 (0.27-13.78) |
| Anterior Cingulate Cortex | 15282 | 9 | 1698 (377-3324) | 3.31 (0.23-15.55) |
| Caudate | 15276 | 6 | 2546 (830-5167) | 2.14 (0.43-5.55) |
| Cerebellar Hemisphere | 15301 | 12 | 1275.08 (182-5156) | 2.48 (0.03-12.5) |
| Cerebellum | 15484 | 14 | 1106 (152-3981) | 2.74 (0.09-16.21) |
| Cortex | 15516 | 12 | 1293 (286-2985) | 3.30 (0.05-18.42) |
| Frontal Cortex | 15442 | 13 | 1188 (379-3133) | 2.89 (0.04-11.66) |
| Hippocampus | 15161 | 8 | 1895 (501-4012) | 3.98 (0.08-10.57) |
| Hypothalamus | 15500 | 7 | 2214 (814-4041) | 3.01 (0.5-8.22) |
| Nucleus Accumbens | 15264 | 5 | 3053 (530-6601) | 3.06 (0.34-6.49) |
| Putamen | 15015 | 6 | 2503 (428-6142) | 3.66 (0.32-13.28) |
| Spinal Cord | 15210 | 9 | 1690 (218-4003) | 5.22 (0.17-17.66) |
| Substantia Nigra | 15086 | 10 | 1509 (313-2994) | 4.20 (0.11-11.93) |

**Supplementary table 7. GTEx age-targeted GCNs modules features per brain region.** The GTEx age-targeted GCNs were created using a ratio of appearance of eight for all the brain regions. Enrichment was calculated according to the methods section.

| **Brain region** | **TGCN size** | **Number of modules** | **Module size (mean, min, max)** | **Enrichment (mean, min, max)** |
| --- | --- | --- | --- | --- |
| Amygdala | 330 | 7 | 47.14 (20-90) | 0.77 (0.1-2.27) |
| Anterior Cingulate Cortex | 530 | 12 | 44.17 (10-90) | 4.18 (0-26.46) |
| Caudate | 650 | 16 | 40.62 (10-90) | 6.20 (0.11-53.56) |
| Cerebellar Hemisphere | 500 | 21 | 23.81 (10-90) | 2.80 (0-40.79) |
| Cerebellum | 630 | 19 | 33.16 (10-80) | 2.83 (0-37.38) |
| Cortex | 670 | 18 | 37.22 (10-90) | 3.98 (0-57.97) |
| Frontal Cortex | 570 | 18 | 31.67 (10-90) | 1.26 (0-5.36) |
| Hippocampus | 300 | 7 | 42.86 (10-90) | 13.36 (0.41-54.59) |
| Hypothalamus | 100 | 3 | 33.33 (20-50) | 11.97 (0-28.99) |
| Nucleus Accumbens | 400 | 13 | 30.77 (10-60) | 3.53 (0-41.03) |
| Putamen | 560 | 17 | 32.94 (10-70) | 5.49 (0.04-33.32) |
| Spinal Cord | 70 | 2 | 35.00 (10-60) | 2.64 (0-4.03) |
| Substantia Nigra | 230 | 5 | 46.00 (30-70) | 8.94 (0-29.53) |

**Supplementary table 8. GTEx age-targeted seeds relevance in the corresponding WGCNA networks per brain region.**

| **Brain region** | **Module membership of targeted seeds in WGCNA modules** (mean, min, max) | **Module membership (percentile) of targeted seeds in WGCNA modules** (mean, min, max) |
| --- | --- | --- |
| Amygdala | 0.514 (0.214-0.750) | 13.86 (1-47) |
| Anterior Cingulate Cortex | 0.620 (0.392-0.771) | 16.58 (1-40) |
| Caudate | 0.473 (0.161-0.686) | 13.50 (1-42) |
| Cerebellar Hemisphere | 0.513 (0.246-0.703) | 15.29 (1-38) |
| Cerebellum | 0.479 (0.123-0.757) | 16.68 (1-65) |
| Cortex | 0.522 (0.237-0.828) | 10.94 (1-30) |
| Frontal Cortex | 0.603 (0.377-0.824) | 14.39 (1-53) |
| Hippocampus | 0.593 (0.400-0.789) | 16.86 (2-43) |
| Hypothalamus | 0.507 (0.378-0.580) | 12.00 (2-24) |
| Nucleus Accumbens | 0.530 (0.353-0.752) | 16.62 (1-61) |
| Putamen | 0.461 (0.140-0.672) | 11.59 (1-32) |
| Spinal Cord | 0.532 (0.407-0.657) | 9.00 (3-15) |
| Substantia Nigra | 0.496 (0.292-0.818) | 21.40 (2-77) |

**Supplementary table 9. Percentage of WGCNA hubs transcripts that showed a significant overlap in TGCNs per brain region.** We selected the top ten transcripts with highest adjacency values within each WGCNA module (hub transcripts) and checked the overlap with the TGCNs modules from the same brain region.

| **Brain region** | **WGCNA hubs transcripts per module**  **preserved in TGCNs modules** (average %) |
| --- | --- |
| Amygdala | 16.67 |
| Anterior Cingulate Cortex (BA24) | 33.33 |
| Caudate (basal ganglia) | 33.33 |
| Cerebellar hemisphere | 33.33 |
| Cerebellum | 35.71 |
| Cortex | 0.00 |
| Frontal cortex (BA9) | 23.08 |
| Hippocampus | 25.00 |
| Hypothalamus | 0.00 |
| Nucleus accumbens (basal ganglia) | 0.00 |
| Putamen (basal ganglia) | 33.33 |
| Spinal cord (cervical C-1) | 0.00 |
| Substantia nigra | 10.00 |

**Supplementary table 10. Negative controls in TGCNs.** We selected a set of genes that, to the best of our knowledge, are not related to the Alzheimer's disease phenotype and that are minimally expressed in the ROSMAP matrix. These genes have a percentile of expression in the ROSMAP FPKM expression matrix between 1 and 88. We applied 10 LASSO runs to predict each negative control gene using the gene expression matrix of ROSMAP as predictors. We observe that the R^2^ for the test sets is much higher for *APP* (0.965 max) compared to that obtained for the other six genes (0.702 max). In addition, the number of seeds selected by LASSO in all the 10 runs is higher for *APP* (9 seeds) compared to the other six genes (0-2 seeds). Finally, we also estimated the enrichment for each targeted GCN as the sum(-log_10_*P*). In the same line, we observed a higher enrichment for the *APP*-target GCN (1998 max) compared to the enrichment of the other six target GCNs (15.88 max). This table is complementary to supplementary figure 2.

| **Seed name** | **Disease associated** | **Percentile expression** | **LASSO R^2^ test (mean, min, max)** | **Number of seeds** | **Enrichment (mean, min, max)** |
| --- | --- | --- | --- | --- | --- |
| *APP* | Alzheimer’s disease | 99 | 0.952  (0.935 - 0.965) | 9 | 293.91  (1.3 - 1998.46) |
| *BRCA1* | Hereditary breast and ovarian cancer | 1 | 0.47  (0.354 - 0.644) | 0 | 0 (0 - 0) |
| *DMD* | Duchenne muscular dystrophy | 39 | 0.484  (0.414 - 0.568) | 2 | 8.83  (1.79 - 15.88) |
| *F8* | Hemophilia A | 36 | 0.638  (0.56 - 0.702) | 0 | 0 (0 - 0) |
| *FBN1* | Marfan syndrome | 12 | 0.598  (0.469 - 0.7) | 2 | 2.66 (0 - 5.32) |
| *NF1* | Neurofibromatosis | 46 | 0.347  (0.149 - 0.44) | 0 | 0 (0 - 0) |
| *TNNT2* | Cardiomyopathy, Dilated, 1D | 88 | 0.275  (0.186 - 0.393) | 2 | 10.8  (7.32 - 14.28) |

**Supplementary table 11. ROSMAP seed transcripts of the *APP*-TGCN replicate in two alternative AD cohorts.**

The seeds detected in ROSMAP with a minimum ratio of appearance of 8, 9 and 10 replicates in Mayo and MSBB since we found a high *APP* predictivity (see adjusted R^2^). To assess whether those values of R^2^ were better than random chance, we performed a permutation analysis by creating 100,000 *APP*-predicting linear models with same size randomly chosen transcripts both in Mayo and MSBB. P-values were estimated dividing the number of models with random transcripts that showed an adjusted R^2^ higher than the model with the relevant transcripts (fp) by the number of total models created (p).

| **Adjusted R^2^** | **fp** | **p** | **p-value** | **Ratio of appearance (*r*)** | **Dataset** |
| --- | --- | --- | --- | --- | --- |
| 0.8248 | 5955 | 100,000 | 0.05955 | 8 | Mayo |
| 0.7997 | 7761 | 100,000 | 0.07761 | 9 | Mayo |
| 0.7858 | 1591 | 100,000 | 0.01591 | 10 | Mayo |
| 0.8632 | 4 | 100,000 | 0.00004 | 8 | MSBB |
| 0.8459 | 4 | 100,000 | 0.00004 | 9 | MSBB |
| 0.7970 | 63 | 100,000 | 0.00063 | 10 | MSBB |

**Supplementary table 12. Effect of missing seed transcripts in the independent cohort to replicate ROSMAP *APP* seed transcripts.** A permutation test was applied to compare the performance of the model created with the *APP* seed transcript with the models created (1) removing each seed individually; (2) removing sets of five seeds (10,000 simulations); (3) removing sets of 10 seeds (10,000 simulations). This strategy was applied for Mayo and MSSB cohorts separately. The R^2^ for each set of models and each cohort is reported in this table (mean, minimum and maximum R^2^).

| **Cohort** | **All seeds (R^2^)** | **Removing one seed each time (R^2^)** | **Removing sets of five seeds (R^2^)** | **Removing sets of 10 seeds (R^2^)** |
| --- | --- | --- | --- | --- |
| Mayo | 0.825 | 0.822 (0.811-0.826) | 0.811 (0.754-0.83) | 0.791 (0.697-0.829) |
| MSBB | 0.863 | 0.86 (0.841-0.864) | 0.844 (0.767-0.867) | 0.814 (0.653-0.864) |

**Supplementary table 13. Summary table of the ROSMAP *APP*-TGCN modules.** This TGCN was created with the seed transcript with a ratio of appearance of eight.

| **Seed name** | **Module size** | **Neuro status p-value** | **Cell type enriched** | **GO stats** | **GO stats corrected by module size** | **Most significant terms** | **Replicated in Mayo**  **(P-value)** | **Replicated in MSBB (P-value)** |
| --- | --- | --- | --- | --- | --- | --- | --- | --- |
| *PPP2CA* | 30 | 0.034 |  | 159.766 | 5.326 | Neuronal System, synapse, neuron projection | 4.48^.^10^-5^ | 1.39^.^10^-15^ |
| *ATP1B1* | 40 | 0.038 | Dopaminergic (*P*<4.552^.^10^-3^) | 184.706 | 4.618 | synapse, Neuronal System, cell junction | 2.76^.^10^-9^ | 1.96^.^10^-10^ |
| *PRKAR1A* | 10 | 0.034 |  | 2.381 | 0.238 | protein kinase A catalytic subunit binding | 7.52^.^10^-8^ | 7.28^.^10^-6^ |
| *TPPP* | 70 | 0.275 |  | 121.605 | 1.737 | nervous system development, Protein-protein interactions at synapses, Neuronal System | 0.536 | 0.377 |
| *FBXO11* | 50 | 0.0962 |  | 128.937 | 2.579 | synaptic signaling, Glutamatergic synapse, Long-term potentiation | 4.1^.^10^-4^ | 1.2^.^10^-6^ |
| *SERINC3* | 60 | 0.034 |  | 18.817 | 0.314 | Renin secretion, Synaptic vesicle cycle, PKA activation | 3.56^.^10^-8^ | 5.96^.^10^-16^ |
| *ABCD3* | 50 | 0.275 | Astrocyte (*P*<1.083^.^10^-3^), Darmanis astrocytes (*P*<6.773^.^10^-3^) | 33.01 | 0.66 | plasma membrane, cell periphery, leukemia inhibitory factor receptor activity | 0.425 | 0.312 |
| *PTAR1* | 30 | 0.034 |  | 29.98 | 0.999 | leukemia inhibitory factor receptor activity, TEAD-YAP complex, oncostatin-M receptor activity | 3.18^.^10^-7^ | 2.08^.^10^-15^ |
| *FBXO9* | 20 | 0.034 |  | 9.385 | 0.469 | COPI-independent Golgi-to-ER retrograde traffic, dynein heavy chain binding, Amplification of signal from unattached kinetochores via a MAD2 inhibitory signal | 2.15^.^10^-3^ | 0.0572 |
| *YWHAE* | 20 | 0.0128 |  | 25.299 | 1.265 | regulation of cellular component organization, Oocyte meiosis, aminophospholipid flippase activity | 1.89^.^10^-4^ | 8.88^.^10^-7^ |
| *SCN8A* | 10 | 0.156 |  | 107.966 | 10.797 | Focal tonic seizure, monoatomic ion channel complex, axon | 8.97^.^10^-4^ | 0.205 |
| *ZFPM1* | 10 | 0.025 |  | 1.302 | 0.13 | Extrapulmonary tuberculosis | 0.102 | 0.0779 |
| *DEAF1* | 20 | 0.0279 |  | 19.743 | 0.987 | RNA biosynthetic process, AKT phosphorylates targets in the cytosol, Spinocerebellar ataxia | 1 | 0.577 |
| *ASTE1* | 10 | 0.0128 |  | 1.301 | 0.13 | Golgi-associated vesicle lumen | 0.102 | 0.257 |
| *RPL36* | 10 | 0.131 |  | 234.365 | 23.437 | Eukaryotic Translation Elongation, cytosolic ribosome, structural constituent of ribosome | 2.15^.^10^-3^ | 0.148 |
| *CEP250* | 80 | 0.804 |  | 10.039 | 0.125 | positive regulation of cellular component biogenesis, Ubiquitin mediated proteolysis, Regulation of PLK1 Activity at G2/M Transition | 1 | 1 |
| *GNAI1* | 80 | 0.101 |  | 51.682 | 0.646 | phosphatidylinositol-3,5-bisphosphate 3-phosphatase activity, phosphatidylinositol-3-phosphate phosphatase activity, phosphatidylinositol-3,5-bisphosphate phosphatase activity | 2.73^.^10^-2^ | 6.6^.^10^-4^ |
| *NCL* | 30 | 0.034 |  | 110.776 | 3.693 | mRNA metabolic process, ribonucleoprotein complex, regulation of mRNA metabolic process | 5.09^.^10^-6^ | 5.66^.^10^-3^ |
| *HNRNPA2B1* | 60 | 2.13e-05 |  | 329.001 | 5.483 | RNA metabolic process, nucleic acid metabolic process, gene expression | 3.16^.^10^-4^ | 1.83^.^10^-15^ |
| *FRZB* | 30 | 0.0962 | Cortex Endothelial Neuroexpresso (*P*<0.0349), Mural (*P*<7.761^.^10^-4^) | 23.443 | 0.781 | anatomical structure morphogenesis, Mydriasis, circulatory system development | 0.0594 | 0.0272 |
| *NFASC* | 70 | 0.000702 | Oligodendrocyte (*P*<3.899^.^10^-4^) | 149.584 | 2.137 | cytoskeletal protein binding, axon, neuron projection | 4.51^.^10^-4^ | 1.03^.^10^-11^ |
| *DGCR6L* | 10 | 0.0128 |  | 8.934 | 0.893 | actin binding, cell cortex, Bulbar palsy | 0.271 | 3.71^.^10^-3^ |
| *SCAF4* | 40 | 0.804 |  | 5.052 | 0.126 | Regulation of actin cytoskeleton, Cell death signalling via NRAGE, NRIF and NADE, p75 NTR receptor-mediated signalling | 0.347 | 0.257 |
| *RPS10* | 20 | 0.555 |  | 1998.461 | 99.923 | Eukaryotic Translation Elongation, cytosolic ribosome, Viral mRNA Translation | 1.1^.^10^-6^ | 3.5^.^10^-9^ |
| *PLPP5* | 20 | 0.467 |  | 1.462 | 0.073 | Role of phospholipids in phagocytosis | 0.0168 | 0.257 |
